# Supplementary material for: Repair protocols for indirect monolithic restorations: a literature review
Source: PeerJ. 2024 Feb 21;12:e16942. doi: 10.7717/peerj.16942 (PMC10893862; doi:10.7717/peerj.16942)
Supplement: Supplemental Information 1 [file peerj-12-16942-s001.docx]

| Table S1. Primary studies of repair protocols of resin-based, glass ceramic, and zirconia materials for indirect restorations | | | | |
| --- | --- | --- | --- | --- |
| Resin-based materials | | | | |
| Study | **Material** | **Protocols** | **Outcome** | **Findings** |
| (Stawarczyk, Krawczuk & Ilie, 2015) | Lava Ultimate  Direct resin composite:  Grandio SO  Arabesk Top | All specimens were thermocycled (10,000 cycles).   1. 30 µm Al_2_O_3_ + SiO_2_ (Cojet) for 10 s with water irrigation; 2. 30 µm Al_2_O_3_ + SiO_2_ (Cojet) for 10 s; 3. Cimara grinding (silicon carbide with 10,000 rpm);   Half of the samples were etched with 34% phosphoric acid. Then, subdivided into five different adhesive systems that were tested: none (control); Futurabond U; One Coat Bond; Scotchbond Universal; and Visio. link. | Tensile bond strength test | Air abrasion with Al_2_O_3_+SiO_2_ produced superior TBS compared to grinding of the surface before repair. The tested adhesive systems proved to be necessary intermediary agents for repairing aged CAD/CAM RNC substrates, while visio.link and Scotchbond Universal performed slightly better than Futurabond U. |
| (Wiegand et al., 2015) | Lava Ultimate*  Ambarino high-class*  Direct resin composite:  Filtek Supreme XTE | All samples were first thermocycled (5,000 cycles).   1. Adhesive only (OptiBond FL); 2. 40 µm diamond disc at 1.3 N for 8 s; 3. 50 µm Al_2_O_3_ air-abrasion for 10 s; 4. 30 µm Al_2_O_3_+SiO_2_ (Cojet) for 10 s + Silane (Monobond Plus).   All samples were thermocycled for 5,000 cycles. | Shear bond strength test | For Lava Ultimate: Cojet air-abrasion = Al_2_O_3_ air-abrasion = Diamond bur > adhesive only.  For Ambarino high-class: Cojet air-abrasion > Al_2_O_3_ air-abrasion > Diamond bur = Adhesive only. |
| (Güngör et al., 2016) | Lava Ultimate*  Cerasmart*  Direct resin composite:  Clearfil Majesty Esthetic | Specimens were first divided into non-thermocycled, cycled (10,000 cycles) before the repair or cycled after.   1. No treatment – Control; 2. 50 µm Al_2_O_3_ for 10 s; 3. Dry grinding with a coarse diamond bur (125 µm); 4. 9% HF for 60 s + Silane. | Surface roughness;  Shear bond strength test | For surface roughness: grinding > air-abrasion > HF + Silane = Control  For Lava Ultimate independently of aging: HF + Silane = Grinding = Air-abrasion > Control  For Cerasmart:  - non-thermocycled: HF + Silane > Air-abrasion = Grinding = Control  - thermocycled after: Grinding = HF + Silane = Air-abrasion ≥ Control  - thermocycled before: Grinding = Air-abrasion ≥ HF + Silane = Control  Thermocycling decreased in general the shear bond strength of the materials. |
| (Subaşı & Alp, 2017) | Lava Ultimate*  Direct resin composite: Filtek Z550 | Half of the specimens were thermocycled (5,000 cycles).   1. Adhesive (Single Bond Universal) – Control; 2. 50 µm Al_2_O_3_ for 20 s; 3. 30 µm Al2O3+SiO2 (Cojet) for 20 s.   Half of the samples were tested after 24h while the other half were thermocycled (5,000 cycles). | Surface roughness;  Shear bond strength test | Air-abrasion increased the surface roughness.  For the non-aged specimens, no difference was found among the surface treatments. Regarding the aged ones, air-abrasion resulted in a higher bond strength than the control group. |
| (Loomans et al., 2017) | Lava Ultimate  Clearfil Estenia  Direct resin composite:  Filtek Supreme XTE | Grinding with a #150-grit silicon carbide paper for 10 s.   1. Adhesive (Scotchbond Multipurpose) only – Control; 2. 30 µm Al_2_O_3_+SiO_2_ (Cojet); 3. 30 µm Al_2_O_3_+SiO_2_ (SilJet); 4. 30 µm Al_2_O_3_+SiO_2_ (SilJet Plus); 5. 50 µm Al_2_O_3_; 6. 9.6% HF for 10 s.   All with or without silane and thermocycling (10,000 cycles). | Microshear bond strength test (rectangular specimens) | For Lava Ultimate: SilJet Plus = SilJet = Al_2_O_3_ = Cojet = 9.6% HF = Only adhesive  For Clearfil Estenia: 9.6% HF > all others.  Silane did not influence the bond strength.  Aging decreased the bond strength only for Clearfil Estenia. |
| (Tatar & Ural, 2018) | Lava Ultimate*  Direct resin composite:  Filtek Z250 | 1. No surface treatment – Control; 2. 9.5% HF; 3. 9.5% HF + Silane (Porcelain Primer); 4. 30 µm Al_2_O_3_+SiO_2_ + Silane (Porcelain Primer) | Surface roughness;  Shear bond strength test | For surface roughness and bond strength: Air-abrasion > HF = HF + Silane > Control |
| (Üstün, Büyükhatipoğlu & Seçilmiş, 2018) | Lava Ultimate*  Direct resin composite:  Tetric N Ceram for Ceramic repair group;  Filtek Z250 for Clearfil Repair group. | All specimens received the first 50 µm Al_2_O_3_.   1. Ceramic repair: 37% phosphoric acid for 5 s + Silane + Adhesive (Heliobond); 2. Clearfil Repair: 37% phosphoric acid for 5 s + Primer (Clearfil SE Bond Primer); 3. mixed 1:1 with and adhesive (Porcelain Bond Activator) + Adhesive (Clearfil SE Bond).   All specimens were aged (5,000 cycles). | Shear bond strength test | No difference was found between the repair systems used. |
| (Flury, Dulla & Peutzfeldt, 2019) | Paradigm MZ100*  Lava Ultimate*  Direct resin composite:  Filtek Z250 | 1. Cojet Prep + 25 µm Al_2_O_3_ + Silane + Adhesive (OptiBond FL); 2. Cojet Prep + 25 µm Al_2_O_3_ + Adhesive (Scotchbond Universal).   Baseline (24h) and after aging (1 year in water). | Shear bond strength | For both materials, the surface treatments performed similarly. |
| (Arpa et al., 2019) | Lava Ultimate*  Direct resin composite:  Filtek Supreme XTE | All samples were first thermocycled (10,000 cycles).   1. 30 µm Al_2_O_3_+SiO_2_ (Cojet) + adhesive (Scotchbond Universal); 2. 30 µm Al_2_O_3_+SiO_2_ (Cojet) + silane (ESPE Sil) + adhesive (Adper Scotchbond 1 XT); 3. 30 µm Al_2_O_3_+SiO_2_ (Cojet) + silane (Monobond Plus) + adhesive (Adper Scotchbond 1 XT); 4. 30 µm Al_2_O_3_+SiO_2_ (Cojet) + adhesive (Adper Scotchbond 1 XT); 5. 27 µm Al_2_O_3_ + adhesive (Scotchbond Universal); 6. 27 µm Al_2_O_3_ + silane (ESPE Sil) + adhesive (Adper Scotchbond 1 XT); 7. 27 µm Al_2_O_3_ + silane (Monobond Plus) + adhesive (Adper Scotchbond 1 XT); 8. 27 µm Al_2_O_3_ + adhesive (Adper Scotchbond 1 XT); 9. Adhesive only (Scotchbond Universal); 10. Adhesive only (Adper Scotchbond 1 XT) | Nanoleakage  Microtensile bond strength test | Air-abrasion with Cojet or alumina is essential for repairing aged Lava Ultimate restorations. Using a universal adhesive effectively increases repair bond strength and provides better sealing. |
| (Demirel & Baltacıoğlu, 2019) | Lava Ultimate  Cerasmart  Shofu Block HC  Direct resin composite:  Clearfil Majesty Esthetic | All samples were thermocycled (5,000 cycles), grounded with a coarse-flat diamond bur with two back-and-forths for 3 s and air-abraded with 30 µm Al_2_O_3_+SiO_2_ (Cojet) for 10 s.   1. Silane (Porcelain Primer) + Adhesive (Adper Single Bond-2) – Control; 2. Adhesive (Single Bond Universal); 3. Adhesive (All Bond Universal); 4. Adhesive (Clearfil Universal);   All specimens were thermocycled (5,000 cycles). | Microshear bond strength test | For Lava Ultimate: no difference was found among the tested surface treatments.  For Shofu Block HC: no difference was found among the tested surface treatments.  For Cerasmart: Clearfil Universal and All Bond Universal resulted in higher bond strength compared to the other tested groups. |
| (Şişmanoğlu et al., 2020) | Lava Ultimate*  Direct resin composite:  Filtek Ultimate Flowable Restorative | 1. Adhesive (Single Bond Universal) only – Control; 2. 9% HF (60 s) + adhesive; 3. 50 µm Al_2_O_3_ + adhesive; 4. 30 µm Al_2_O_3_+SiO_2_ (Cojet) + adhesive;   All with or without silane. | Surface roughness;  Microshear bond strength | Both air-abrasion surface treatments increased the surface roughness compared to HF and control.  For bond strength: Al_2_O_3_+SiO_2_ > Al_2_O_3_> HF > Adhesive. The application of silane increased the bond strength for all groups. |
| (Gul & Altınok-Uygun, 2020) | Lava Ultimate*  Cerasmart*  Direct resin composite:  Tetric EvoCeram | 1. Adhesive; 2. 34.5% phosphoric acid + adhesive; 3. 30 µm Al_2_O_3_+SiO_2_ (Cojet) + adhesive; 4. Z-Primer Plus - 9.5% HF for 90 s + primer; 5. GC Repair - Ceramic Primer II + G-Premio Bond; 6. Cimara System: grinding bur + silane + opaquer liquid; 7. Porcelain Repair : 9% HF + silane + adhesive; 8. Clearfil Repair System: 40% phosphoric acid + mix of bond with activator + adhesive.   All samples were aged (5,000 cycles). | Microtensile bond strength | The Cimara System, Porcelain Repair, and Clearfil Repair showed significantly higher bond strength compared to other systems. However, all tested repair systems resulted in clinically acceptable bond strength. |
| (Bahadır & Bayraktar, 2020) | Lava Ultimate*  Direct resin composite:  Filtek Ultimate | Half of the samples were thermocycled (5,000 cycles).   1. Adhesive; 2. Silane + Adhesive; 3. Diamond bur 107 – 181 µm grain size for 4 s + adhesive; 4. Diamond bur 107 – 181 µm grain size for 4 s + silane + adhesive; 5. Er,Cr:YSGG laser with a wavelength of 2780 nm for 20 s (3 W, 20 Hz, air level 60%, and water level 50%) + adhesive; 6. Er,Cr:YSGG laser + silane + adhesive.   All samples were aged (10,000 cycles). | Microtensile bond strength  Color change  Surface roughness | Control groups (without grinding or laser application) resulted in worse adhesive behavior than the others. Grinding with diamond bur and the laser application seems like proper surface treatment method for repair. |
| (Sismanoglu et al., 2020) | Brilliant Crios  Lava Ultimate  Shofu Block HC  Direct resin composite:  Filtek Ultimate Universal | All samples were aged (5,000 cycles) and grounded with a #320-grit SiC disk.   1. Adhesive only (Clearfil Universal Bond); 2. Adhesive only (Prime&Bond Universal); 3. Adhesive only (Single Bond Universal); 4. 9% HF for 60 s + adhesive (Clearfil Universal Bond); 5. 9% HF for 60 s + adhesive (Prime&Bond Universal); 6. 9% HF for 60 s + adhesive (Single Bond Universal); 7. 30 µm Al_2_O_3_+SiO_2_ (Cojet) for 15 s + adhesive (Clearfil Universal Bond); 8. 30 µm Al_2_O_3_+SiO_2_ (Cojet) for 15 s + adhesive (Prime&Bond Universal); 9. 30 µm Al_2_O_3_+SiO_2_ (Cojet) for 15 s + adhesive (Single Bond Universal). | Surface roughness;  Microtensile bond strength test | HF acid etching is not recommended for materials composed of an increased composite resin content.  Micromechanical retention with airborne-particle abrasion or tribochemical silica airborne-particle abrasion is essential for repairing aged resin nanoceramics.  The use of a universal adhesive alone reported promising results. |
| (Veríssimo et al., 2020) | Lava Ultimate*  Direct resin composite:  Filtek Z350 | Half of the samples were *in situ* aged.   1. Diamond bur for 20 s + adhesive (Single Bond Universal); 2. Diamond bur for 20 s + Silane (Prosil) + Adhesive (Amber); 3. 10% HF (20 s) + Silane (Prosil) + Adhesive (Amber); 4. 30 µm Al_2_O_3_+SiO_2_ (Cojet) for 20 s + Silane (Prosil) + Adhesive (Amber); 5. Al_2_O_3_ + Silane (Prosil) + Adhesive (Amber);   All samples were thermocycled (10,000 cycles). | Shear bond strength test;  Colony forming units;  Surface roughness | For bond strength: Diamond bur+Adhesive = Diamond bur+Silane+Adhesive = Air-abrasion Al_2_O_3_+Silane+Adhesive ≥ HF+Silane+Adhesive = Air-abrasion Cojet+Silane+Adhesive |
| (Moura et al., 2020) | Lava Ultimate*  Direct resin composite: Filtek Z350 | Half of the samples were aged (6 months).   1. 50 µm Al_2_O_3_ for 20 s + adhesive (Scotchbond Universal); 2. 30 µm Al_2_O_3_+SiO_2_ (Cojet) for 20 s + adhesive (Scotchbond Universal). | Shear bond strength test | For non-aged and aged samples: Air-abrasion with Al_2_O_3_ = Air-abrasion with Al_2_O_3_+SiO_2_ |
| (Bayraktar, Arslan & Demirtag, 2021) | Lava Ultimate*  Direct resin composite: Filtek Z250 | All samples were aged (10,000 cycles).   1. Coarse diamond bur 107–181 μm by 15 movements in each axis for 15 s. 2. 9% HF (60 s); 3. Nd: YAG laser 1,064 nm wavelength (3 W output power, a repetition rate of 20 Hz, and a pulse duration of 200 μs for 20 s with air/water flow); 4. Er:YAG laser 2,940 nm wavelength (3 W output power, a repetition rate of 20 Hz, and a pulse duration of 300 μs for 20 s with 60% air and 50% water flow); 5. ErCr: YSGG laser 2,780 nm wavelength (3 W output power, a pulse repetition rate of 20 Hz, and a pulse duration of 140 μs for 20 s with 60% air and 50% water flow);   All specimens received silane (Ceramic Primer II) for 60 s + adhesive (Single Bond Universal). After the repair, the specimens were thermocycled (10,000 cycles). | Microtensile bond strength test | Bur grinding and 9% HF were the surface treatments with the highest bond strength. Er:YAG laser, Er:Cr:YSGG laser, and Nd:YAG laser resulted in a lower bond strength respectively in this order. |
| (Bayazıt, 2021) | Lava Ultimate*  Cerasmart*  Direct resin composite:  G-aenial Universal Flo | All samples were aged (5,000 cycles).   1. Control (no treatment) 2. Adhesive only (Single Bond Universal); 3. 9.5% HF (60 s); 4. 50 µm Al_2_O_3_ for 20 s; 5. 9.5% HF + primer (Ceramic Primer II) + adhesive (G-Premio BOND); 6. 50 µm Al_2_O_3_ for 20 s + adhesive (Single Bond Universal); 7. 50 µm Al_2_O_3_ for 20 s + primer (Ceramic Primer II) + adhesive (G-Premio BOND). | Microtensile bond strength test | For Lava Ultimate: Air-abrasion + adhesive = HF + adhesive > HF = HF+PR+Adhesive = Air-abrasion = Adhesive only = Air-abrasion+PR+Adhesive  For Cerasmart: HF+Adhesive = Air-abrasion+Adhesive = HF = Adhesive only = HF+Primer+Adhesive > Air-abrasion = Air-abrasion+Primer+Adhesive |
| (Arkoy & Ulusoy, 2022) | Grandio Blocs*  Shofu Block HC*  Lava Ultimate*  Direct resin composite:  Grandio SO | All samples were aged (10,000 cycles).   1. Only adhesive (Single Bond Universal) – Control; 2. Coarse diamond bur for 15 s with 15 movements in each axis; 3. 50 µm Al_2_O_3_ for 20 s; 4. Er,Cr: YSGG-2W laser irradiation (2W, 140 ms pulse duration and a repetition rate of 10 Hz with 55% water and 65% air for 20 s); 5. Air-abrasion + laser;   All specimens received the application of an adhesive (Single Bond Universal). | Microtensile bond strength test | For Grandio Blocs: The combination of air-abrasion + laser resulted in a higher bond strength, similar to the other surface treatments, than the control group.  For Shofu Block HF: Combined = Laser = Control > Bur grinding = Air-abrasion  For Lava Ultimate: Bur grinding = Air-abrasion = Combined = Laser > Control |
| Glass-ceramic materials | | | | |
| Study | **Material** | **Protocols** | **Outcome** | **Findings** |
| (Colares et al., 2013) | IPS e.max CAD (LD)  Direct resin composite:  Filtek Z250 | 1. G1: HF 9.5% (20 s) and silanated surface was dried at room temperature (23 °C); 2. G2: HF 9.5% (20 s) and the silanated surface was dried with warm air at 45 ± 5 °C; 3. G3: 50 μm Al2O3 for 5 s and the silanated surface was dried with warm air at 45 ± 5 °C; 4. G4: sandblasting with 50 μm aluminum oxide particles (5 s) and silanated surface was dried with room-temperature air.   After, adhesive was applied to all groups. | Microtensile bond strength | Hydrofluoric acid and the application of silane can be used as an alternative to repair ceramic restorations with composite resin. Surface pretreatment with sandblasting should be avoided. |
| (Erdemir et al., 2014) | IPS e.max CAD (LD)  Direct resin composite:  Self-adhering, flowable resin composite - Vertise Flow | 1. Group C-untreated (control): No treatment was applied to the repair surfaces; 2. Tribochemical silica coating: 30 µm silica-coated alumina particles air-abrasion at 2.5 bar air pressure from a distance of 10 mm (10 s); 3. 9.6% HF (20 s); 4. Er:YAG laser wavelength of 2.940 nm, a power setting of 300 mJ, 20 Hz, average power output of 6 W delivered to the repair surface with a 10-mm-long and 1-mm-diameter sapphire tip (20 s); 5. Group DB-diamond bur: surface ground by using a red band 30-µm-grit-size round-ended cylindrical diamond bur (10 s)   Silane was applied afterward in all groups except control | Surface roughness  Shear bond strength | Hydrofluoric and Diamond bur grinding produced the highest surface roughness.  Hydrofluoric, Diamond bur grinding, and Er:YAG laser produced the highest bond strength values.  Self-adhering flowable resin composite used as repair material exhibited unsatisfactory bond strength irrespective of the surface pretreatments used. |
| (Huang, Wang & Gao, 2013) | IPS Empress 2 (LD)  Direct resin composite:  Ceram·X Mono | Two general treatments   1. Diamond abraded, ultrasonic cleaned with distilled water (5 min) and air dried. 2. Coated by 30μm silica-modified Al_2_O_3_ particles at a distance of less than 1 cm and a pressure of 100psi.   Combined with the following treatments   1. Etched by 37% phosphoric acid (60 s), water cleaned for above 20 s under high pressure, and air dried.; 2. Etched by 9.6% HF (30 s), water cleaned for above 20 s under high pressure and air dry; 3. Silanised the surfaces with Calibra silane (60 s) and air dry; 4. –: no treatment.   All specimens received adhesive application and were subjected to 5000 thermocycles, 5°C to 55ºC. | Microtensile bond strength | Before applying the bonding agents on the lithium disilicate-based ceramic, etching by HF or a combined application of diamond abrasion and silanization might be recommended for lithium disilicate-based ceramic repair. |
| (Duzyol et al., 2016) | Cerec Blocs (FEL)  IPS e.max CAD (LD)  Direct resin composite:  Filtek Z550 | 1. Control, roughened with bur (B); 2. Roughened with bur and 5% HF (5 min); 3. Roughened with bur and sandblasting using A1_2_O_3_ with a particle size of 50 µm (10 s), at a 5 mm distance from the specimen (SB); 4. Roughened with bur and CoJet sand was applied (10 s) from a 5 mm distance (C)   After surface treatments on each subgroup, a silane and a bonding system were applied in accordance with the manufacturers’ instructions. | Microtensile bond strength | HF etching was the best lithium disilicate surface treatment. Roughening with bur and sandblasting was not appropriate for lithium disilicate.  The surface treatments did not improve the bond strength in feldspar and resin nano ceramics when compared with their own control groups. |
| (Al-Thagafi, Al-Zordk & Saker, 2016) | IPS e.max Suprinity (ZLS);  IPS e.max CAD (LD).  Direct resin composite:  Tetric Evo Ceram | 1. No treatment – Control; 2. HF 5% (60 s) + silane (Monobond-S) for 60 s, air drying; 3. HF 5% (60 s) + silane (Monobond-S) for 60 s, air drying, Heliobond for 20 s; 4. 30 µm Al_2_O_3_+SiO_2_ (Cojet) + silane (Monobond-S) for 60 s. | Microtensile bond strength | Repair bond strength to zirconia-reinforced lithium silicate ceramics and lithium-disilicate glass ceramic could be improved when ceramic surfaces are sandblasted with CoJet sand followed by silanization or the use of HF, silane, and adhesive. |
| (Ataol & Ergun, 2018) | IPS e.max CAD (LD);  IPS e.max Suprinity (ZLS).  Direct resin composite:  Clearfil Majesty | 1. HF 9% (90 s) + ultradent silane + Clearfil Universal Bond; 2. 50 μm Al2O3 (20 s) + ultradent silane + Clearfil Universal Bond; 3. Er,Cr:YSGG laser with 3 W, air (50%) and water (1%) cooling at 2.94 µm wavelength, pulse repetition of 50 Hz, pulse duration of 140 µs + ultradent silane + Clearfil Universal Bond; 4. HF 9% (90 s) + Clearfil Ceramic Primer; 5. 50 μm Al2O3 (20 s) + Clearfil Ceramic Primer; 6. Er,Cr:YSGG laser + Clearfil Ceramic Primer; 7. Ultradent silane + Clearfil Universal Bond – Control; 8. Clearfil Ceramic Primer – Control. | Shear  bond strength | 1. Alumina blasting and HF provided satisfactory repair bond strengths. 2. HF was more effective than alumina blasting and Er,Cr:YSGG laser irradiation. 3. Er,Cr:YSGG laser irradiation could not be regarded as an effective surface treatment for roughening each CAD/CAM ceramic surface to establish better bond strength with a resin composite.   A single-stage bonding agent may be preferred to silane/bond application for repairing the fractured CAD/CAM since it not only saves time but it also is easy to use and apply. |
| (Ebrahimi Chaharom et al., 2018) | IPS e.max Press (LD)  Direct resin composite:  P90 silorane-based resin composite | 1. No surface treatment – Control; 2. HF 9.5% (60 s); 3. Nd:YAG laser with power setting = 4.5 W, energy parameter = 30 mJ, repetition rate = 15 Hz and wavelength = 1.064 μm. Tip perpendicular to the ceramic surface at 1 mm distance for 60 s; 4. Nd:YAG laser with power setting = 6 W, energy parameter = 300 mJ, repetition rate = 20 Hz, wavelength = 1.064 μm. Tip perpendicular to the ceramic surface at 1 mm distance for 60 s; 5. Er,Cr:YSGG laser with power setting = 1.5 W, energy parameter = 300 mJ, wavelength = 2.78 nm. Tip perpendicular to the ceramic surface at 1 mm distance for 60 s; 6. Er,Cr:YSGG laser with power setting = 6 W, energy parameter = 300 mJ, wavelength = 2.78 nm. Tip perpendicular to the ceramic surface at 1 mm distance for 60 s.   After, silane and adhesive were applied to all groups. | Shear  bond strength | The use of HF proved the most effective surface preparation technique to increase the repair bond strength between lithium disilicate glass ceramic and silorane-based composite resin. |
| (Karci et al., 2018) | IPS empress CAD (LEU)  IPS e.max CAD (LD)  Direct resin composite:  Tetric N Ceram  SuperFlow | All specimens: HF 5% for 20 s - e.max CAD and 60 s for - Empress CAD   1. Ceramic repair:   Monobond S for 60 s + Heliobond for 10 s + Tetric Evoceram   1. SuperFlow:   Only self-adhesive resin application.  All specimens were then aged for 5000 thermal cycles between 5°C and 55°C, with dwell and transfer times of 20 s. | Shear  bond strength | Both repair strategies were similar within the tested materials. |
| (Üstün, Büyükhatipoğlu & Seçilmiş, 2018) | IPS e.max CAD (LD)  Vita Suprinity (ZLS)  Direct resin composite  Filtek Z250  Tetric N Ceram | Specimens were subjected to 5000 thermal cycles between 5 and 55°C with dwell and transfer times of 20 seconds. After, HF 9% (20 s).   1. Ceramic Repair: Monobond S (60 s), and then Heliobond was applied and light-cured for 10 seconds, then Heliobond adhesive system was applied. 2. Clearfil Repair: Clearfil SE Bond Primer and Porcelain Bond Activator were mixed in a 1:1 ratio and applied for 5 seconds. Thereafter, Clearfil SE Bond was applied and light-cured for 10 seconds. | Shear  bond strength | The two investigated repair systems allow for successful repairs. |
| (AL-Turki et al., 2020) | Vitablocs Mark II (FEL)  IPS e.max Suprinity (ZLS);  Direct resin composite:  Tetric N-Collection | 1. No surface treatment – Control; 2. HF 5% (90 s) 3. APA: 50 µm Al_2_O_3_ for 10 s; 4. TSC: 45 μm Al_2_O_3_+SiO_2_, at 10 mm distance perpendicular to adhesive surface, under 2.8 bar pressure for 20 seconds.   An adhesive system was applied prior to the resin composite in all groups. | Microtensile bond strength | For feldspathic, hydrofluoric acid was superior to other surface treatments.  For zirconia-reinforced lithium silicate, all the treatments produced similar bond strength. |
| (Bayraktar, Arslan & Demirtag, 2021) | Vitablocs Mark II (FEL)  Direct resin composite:  Filtek Z250 | 1. Bur grinding - Grinding with diamond bur with 107–181 μm grain size by 15 horizontal and 15 vertical movements across the specimen under low hand pressure for 15 s; 2. HF 9% (60 s); 3. Nd:YAG laser - 1,064 nm wavelength NY laser with a 300 μm diameter fiber optic tip from 1 mm distance to surface at 3 W output power, a repetition rate of 20 Hz, and a pulse duration of 200 μs for 20 s with air/water flow. 4. Er:YAG laser - 2,940 nm wavelength EY laser with a 900 μm diameter fiber optic tip from 1 mm distance to surface at 3 W output power, a repetition rate of 20 Hz, and a pulse duration of 300 μs for 20 s with 60/50 air/water %flow; 5. ErCr:YSGG laser - 2,780 nm wavelength EY laser with a 600 μm diameter fiber optic tip from 1 mm distance to surface at 3 W output power, a repetition rate of 20 Hz, and a pulse duration of 140 μs for 20 s with 60/50 air/water %flow.   After, an adhesive system was applied in all groups before the resin composite application. | Microtensile bond strength | HF acid etching was still the most proper surface treatment technique for repairing feldspathic ceramic restorations. Lasers are not recommended for surface treatment procedures with feldspathic ceramics. |
| (Kilinc, Sanal & Turgut, 2020) | Vita Mark II (FEL)  IPS e.max CAD (LD)  Direct resin composite:  Filtek Z550 | 1. No additional surface treatment was carried out (control); 2. Air abrasion with 50 µm Al_2_O_3_ particles at a distance of 10 mm under 3 bar pressure (20 s); 3. HF 5%, feldspathic ceramic specimens were applied with HF for 60 seconds; while the etching time was 20 seconds for lithium disilicate; 4. Er,Cr: YSGG laser with 2780 nm wavelengths and an 800-μm diameter quartz tip, 2 W power, 20 MHz frequency, and 140 μs pulse period with 60% air and 50% water at a distance of 5 mm (40 s).   A universal adhesive was used in all specimens before resin composite restoration.  Half of the specimens were aged with 5000 thermal cycles between 5 - 55°C with 30 seconds of dwell time and 5 seconds of transfer time for each material. | Shear bond strength | Etching was more effective in achieving durable SBS for both ceramic materials.  Laser irradiation could be considered as an alternative surface treatment method to air abrasion for all tested materials. |
| (Maawadh et al., 2020) | IPS e.max Press (LD)  Direct resin composite:  Clearfil Majesty Esthetic | 1. Laser Irradiated using Er, Cr: YSGG (ECYL) wavelength 2790 nm at 30Hz frequency and power 3.75W, pulse rate of 140µs with a 300µm sapphire tip, MZ8 in a non-contact position in a circular motion for a duration (120 s). Air water ratio was maintained at 65% to 35%; 2. Photodynamic therapy (PDT) using Methylene blue photosensitizer (MBPS) at a concentration of 100mg/L at power 1.5W and wavelength 810nm. Fiber optic tip diameter of 150µm (30 s); 3. 9.6% HF (60 s) – salinization (60 s); 4. Air Abrasion (CoJet) (10 s) at 2bar pressure from a distance of 10 mm - salinization (60 s).   After, an adhesive system was applied in all groups before the resin composite application.  All specimens were subjected to thermocycling, 5°C to 60ºC (number of cycles not provided). | Shear bond strength | Hydrofluoric acid + salinization along with air Abrasion + salinization showed predictable bonding outcomes as compared to other treatments. |
| (Sanal & Kilinc, 2020) | VITA VM 9, VITA VM 13 and VITA VMK 95 (FEL)  IPS e.max Ceram (FLU)  Direct resin composite:  Vertise Flow self-adhesive flowable composite resin, Fusio Liquid Dentin self-adhesive flowable composite resin, Constic self-adhesive flowable composite resin, Filtek Supreme XTE | Thermal cycles for 5,000 cycles between 5°C and 55°C with dwell times of 20 seconds. Afterward, the specimens’ upper surfaces were roughened using a medium-grit diamond rotary cutting instrument, then airborne-particle abraded with 50-μm aluminum oxide particles at a pressure of 2 atm from a distance of 5 mm (5 s). After, specimens were repaired as follows:   1. Vertise Flow - Brush in a thin layer of VF for 20 s and light cure for 20 s. Then, apply VF with a Luer Lock Tip in layers not exceeding 2 mm. Light cure each layer for 20 s. 2. Fusio Liquid Dentin - Brush in a thin layer of FLD for 20 s and light cure for 10 s. Then apply FLD with a Luer Lock Tip in layers not exceeding 2 mm. Light cure each layer for 10 s, and the final layer for 20 s. 3. Constic - Brush in a thin layer of C for 20 s and light cure for 20 s. Then apply C with a Luer Lock Tip in layers not exceeding 2 mm. Light cure each layer for 20 s. 4. BISCO Intraoral Repair Kit + Filtek Supreme (BC + FS) - Apply HF for 60 s. Apply 1 coat of porcelain to the etched surface and allow it to dwell for 30 s. Dry with an air syringe. Apply a thin layer of porcelain bonding resin. Spread the FS to the bonded surface in layers not exceeding 2 mm. Light cure each layer for 20 s. | Color stability  Shear bond strength | All the investigated repair resin composites exhibited ΔE values higher than the clinically acceptable limits for the investigated ceramic veneers.  Self-adhesive composites (except for Vertise Flow) could be used instead of a repair kit with flowable composite for the purpose of repairing chipping fractures of VITA VM 9, VITA VMK 95, and VITA VM 13.  Vertise Flow was insufficient for the purpose of repairing the four investigated ceramics.  All of the investigated repair materials showed low bonding strength with IPS e.max Ceram. |
| (Veríssimo et al., 2020) | Vita Suprinity (ZLS)  Direct resin composite  Filtek Z350 XT | Half of the specimens were subjected to in situ aging in dentures and were worn by patients for 60 days.   1. DbSBU: diamond-bur roughening of the whole surface for 20 s and Single Bond Universal adhesive application. 2. DbSiCa: surface roughening was performed as described above. Then, silane was applied to the whole surface, afterward, the adhesive Ambar was applied. 3. HFSiCa: Etching with 10% HF (20 s), silane application, and adhesive application. 4. CjSiCa: The surface was sandblasted with Cojet (20 s) at a distance of 15 mm with a 2.5 bar pressure. Then, silane and adhesive were applied. 5. SbSiCa: Sandblasting as described previously. After washing and drying, silane and adhesive were applied.   After, an adhesive system was applied in all groups before the resin composite application.  The block-cylinder assemblies were exposed to thermocycling of 10.000 cycles in 30-s alternate baths of 5 and 55 °C previously to shear bond strength test. | Shear bond strength | Acid etching + silanization was the best repair protocol |
| (Ueda et al., 2021) | IPS e.max CAD (LD)  Direct resin composite:  Clearfil AP-X | 1. Monobond Etch & Prime (20 s); 2. K-etchant GEL (15 s) + Clearfil Universal Bond; 3. Bondmer Lightless applied and immediately air-dried with medium air pressure (10 s); 4. K-etchant GEL for 15 s + G-Multi PRIMER applied (20 s), and air-dried (10 s)   Half of the specimens were subjected to 5000 thermal cycles of 5°C and 55°C with a dwell time of 30 s. | Micro-shear bond strength | Monobond Etch & Prime presented high bond stability.  After aging, Bondmer Lightless and Monobond Etch & Prime were similar and superior to the other groups. |
| (Atala & Yeğin, 2022) | Vitablocs Mark II (FEL)  Direct resin composite:  Filtek Z250 | 1. All-Bond - 50 µm Al_2_O_3_ particle abrasion, HF (60 s), Silane, Adhesive; 2. Prime&Bond - Same as above; 3. Clearfil Quick - 50 µm Al_2_O_3_ particle abrasion, Phosphoric acid (5 s), Adhesive 4. Premio Bond - 50 µm Al_2_O_3_ particle abrasion, Silane, Adhesive 5. Optibond XTR - 50 µm Al_2_O_3_ particle abrasion, HF (60 s), Silane, Primer, Adhesive 6. Tokuyama - Airborne particle abrasion, Bonding | Microshear bond strength | Clearfil Quick, Prime&Bond, and Premio Bond presented the best bond strength results. |
| (Höller et al., 2022) | IPS e.max CAD (LD)  Direct resin composite:  Tetric EvoCeram | 1. GBL: Grit blasted with alumina (35 μm at 1 bar pressure (10 s) and a working distance of 4 ± 1 cm) and primed. 2. MEP: Self-etching glass-ceramic primer. 3. NoPT: No surface pretreatment served as a control.   All pretreated surfaces were coated with Heliobond adhesive system.  Controlled relative humidity (RH) and temperature to simulate three different environmental settings:   1. Laboratory conditions (LC, 23°C, 50% RH) 2. Rubber-dam conditions (RC, 30°C, 50% RH) 3. Oral conditions (OC, 32°C, 95 ± 5% RH)   Half of the specimens were subjected to 5000 thermocycles, 5°C to 55ºC. | Tensile bond strength | MEP pretreatment is a possible alternative to GBL for the repair of lithium-disilicate glass ceramics;  Increased humidity significantly reduced the adhesion potential of resin composites to lithium-disilicate glass ceramics pretreated with MEP and GBL;  Minimizing humidity is favorable for the adhesion of resin composite repairs to lithium-disilicate glass ceramic, and the clinical use of a rubber-dam is suggested. |
| (Aladağ & Ayaz, 2023) | Vita Suprinity (ZLS)  Nice block (LD)  Direct resin composite:  Estelite Sigma Quick | 1. No surface treatment applied – Control; 2. Er: YAG laser, wavelength of 2940 nm, 2 W output power, 200 mJ pulse energy, 10 Hz pulses/s, 75 µs pulse length; 3. Nd: YAG laser, wavelength of 1064 nm, 2 W output power, 100 mJ pulse energy, 20 Hz pulses/s, 150 µs pulse length; 4. HF 5% (20 s); 5. HF 5% + Er: YAG laser; 6. HF 5% + Nd: YAG laser; 7. 50 µm Al_2_O_3_ (15 s).   After, an adhesive system was applied in all groups before the resin composite application. | Shear bond strength | For both ceramics, acid etching improved bond strength, especially when combined with laser application. |
| Zirconia materials | | | | |
| Study | **Material** | **Protocols** | **Outcome** | **Findings** |
| (Aladağ & Ayaz, 2023) | Yttrium oxide partially stabilized tetragonal zirconia polycrystal (Y-TZP)- Upcera ST (ZR)  Direct resin composite: Estelite Sigma Quick | 1. No surface treatment – Control;  2. Er: YAG laser, wavelength of 2940 nm, 2 W output power, 200 mJ pulse energy, 10 Hz pulses/s, 75 µs pulse length;  3. Nd: YAG laser, wavelength of 1064 nm, 2 W output power, 100 mJ pulse energy, 20 Hz pulses/s, 150 µs pulse length;  4. HF 5% (20s);  5. HF 5% + Er:YAG laser;  6. HF 5% + Nd:YAG laser;  7. 50 μm Al_2_O_3_ for 15s.  For all specimens: Clearfil Ceramic Primer Plus and Single Bond Universal adhesive. | Shear bond strength test | The use of laser irradiation (Er: YAG and Nd: YAG) depicted the highest bond strength for the zirconia ceramic. HF etching was similar to the absence of treatment (control group). |
| (Greuling et al., 2023) | 5Y-PSZ - Z-CAD® smile (ZR)  Direct resin composite: Estelite sigma Quick | 1. No repaired crown – Control;  2. Repaired crown (only universal adhesive application);  3. Repaired and thermomechanically (1,000,000 cycles of 90 N at 1.2 Hz; 6,000 cycles of thermocycling) aged crown. | Fracture resistance test | The repaired (aged or not) crowns depicted lower values of load to fracture when compared to the crowns without trepanation. |
| (Klaisiri et al., 2022) | Zirconia - Ceramill Zolid HT (ZR)  Direct resin composite:  SimpliShade universal nanohybrid universal | All specimens were air-abraded (50 μm Al_2_O_3_) for 10s.  1. No Primer;  2. One primer application (Clearfil ceramic primer plus);  3. Two primer applications;  4. Three primer applications;  5. Four primer applications;  6. Five primer applications;  For all groups: adhesive agent application (Optibond S). | Shear bond strength test | The group with 3 applications of primer depicted the highest bond strength values, with no difference for 4 and 5 applications. |
| (Ordueri, Ateş & Özcan, 2023) | IPS e.max CAD ZirCad (ZR)  Porcelain - e.max Ceram (FEL)  Direct resin composite:  Clearfil Esthtetic Majesty Composite  Indirect technique:  IPS Empress CAD (LEU) | 1. Zirconia only;  2. Veneered zirconia (70% zirconia + 30% porcelain);  3. Veneered zirconia (50% zirconia + 50% porcelain);  4. Veneered zirconia (30% zirconia + 70% porcelain);  5. 100% porcelain.  Each group was repaired by direct (Clearfil Ceramic Primer and Clearfil SE Bond primer mixture + Clearfil SE BOND adhesive) or indirect technique. | Shear bond strength test | The repair with porcelain veneers (indirect technique depicted higher values of bond strength when compared to the direct resin composite repair, being the techniques similar just when 50% of zirconia and porcelain were considered. |
| *Did not simulate the repaired surface with a diamond bur  HF – Hydrofluoric acid etching; Al_2_O_3_ – air-abrasion with aluminum oxide; Al_2_O_3_+SiO_2_ – tribochemical silica airborne-particle abrasion; FEL - Feldspathic ceramic; LD - Lithium disilicate; ZLS - Zirconia-reinforced lithium silicate; LEU - Leucite glass ceramic; FLU - Fluorapatite ceramic; ZR – Zirconia-based ceramic. | | | | |
