# Supplementary material for: Repair protocols for indirect monolithic restorations: a literature review
Source: PeerJ. 2024 Feb 21;12:e16942. doi: 10.7717/peerj.16942 (PMC10893862; doi:10.7717/peerj.16942)
Supplement: Supplemental Information 2 [file peerj-12-16942-s002.pdf]

10-year randomized trial (RCT) of zirconia-ceramic and metal-ceramic fixed dental prostheses

**Type**	Journal Article
**Author**	Irena Sailer
**Author**	Marc Balmer
**Author**	Jürg Hüsler
**Author**	Christoph Hans Franz Hämmerle
**Author**	Sarah Känel
**Author**	Daniel Stefan Thoma
**Date**	2018-09
**Volume**	76
**Pages**	32–39
**Publication**	Journal of Dentistry
**DOI**	10.1016/j.jdent.2018.05.015
**ISSN**	03005712
**Date Added**	1/26/2024, 8:45:05 AM
**Modified**	1/26/2024, 8:45:05 AM

A Simple Technique to Repair Feldspathic Porcelain Chipping in Screw-retained Implant-supported Prosthesis: A Clinical Technique.

**Type**	Journal Article
**Author**	Lenin Proaño
**Author**	Rebeca K Silva
**Author**	Ariadne Cc Cruz
**Author**	Mutlu Özcan
**Author**	Claudia Âm Volpato
**Abstract**	AIM This clinical technique report aimed to describe a composite resin repair technique performed in an implant-supported prosthesis. BACKGROUND Veneering ceramic fracture or chipping is one of the most frequent clinical failures in dentistry. Therefore, the use of less time- and cost-consuming ceramic repair techniques is helpful in clinical practice. TECHNIQUE Briefly, to treat the ceramic surface, the glaze was removed at the margins of the fracture area, then, air-abrasion and acid-etching were performed. To promote chemical adhesion, a silane coupling agent and adhesive system were applied over the ceramic surface, and the composite resin was applied by incremental technique. Finally, the polish was performed. CONCLUSION In conclusion, the applied composite resin repair of feldspathic porcelain chipping in implant-supported prosthesis was a simple, easy, affordable, and minimally invasive treatment. CLINICAL SIGNIFICANCE The causes of veneer materials failures in metal-ceramic crowns are considered a challenge for the dentist and a problem that displeases patients. Repairs are indicated to prevent cracks from spreading and to prevent the accumulation of biofilm on the damaged surface. Therefore, different repair protocols have been proposed to enhance the esthetic, functionality, and longevity of the implant-supported prosthesis. Additionally, the success of the clinical cases depends on the capability to identify ceramic failures and the ability to indicate/perform the correct repair protocol. Since the described repair technique of the fractured screw-retained implant-supported prosthesis was a simple, easy, affordable, and minimally invasive treatment, with excellent esthetic and masticatory results, it represents an interesting clinical option.
**Date**	2021-01
**Extra**	PMID: 34002718
**Volume**	22
**Pages**	101–104

**Publication**	The journal of contemporary dental practice
**Issue**	1
**ISSN**	1526-3711
**Date Added**	1/26/2024, 8:45:05 AM
**Modified**	1/26/2024, 8:45:05 AM

Academy of Dental Materials guidance on in vitro testing of dental composite bonding effectiveness to dentin/enamel using micro-tensile bond strength (µTBS) approach

**Type**	Journal Article
**Author**	Steve Armstrong
**Author**	Lorenzo Breschi
**Author**	Mutlu Özcan
**Author**	Frank Pfefferkorn
**Author**	Marco Ferrari
**Author**	Bart Van Meerbeek
**Abstract**	Objective An ideal dental adhesive should provide retentive strength, marginal seal, be relatively simple to achieve and demonstrate clinical durability. Future improvements in adhesive bonding to tooth structure require in vitro test methods that provide reliable data for materials development and/or evaluation of experimental variables. The objective of this project was to identify a test method that is relatively easy to perform, repeatable and ultimately useful for predicting clinical outcomes. Methods The Academy of Dental Materials initiated a project to develop and distribute guidance documents on laboratory test methods that are useful for the evaluation of dental adhesives and cements, composite resins and ceramics. Results The dental adhesive sub-group has identified the micro-tensile bond strength test, especially after subjecting the specimens to a durability challenge, as currently the best practical surrogate measure of dental composite restoration retention. Conclusion The following µTBS guidance is meant to aid the researcher in conducting the µTBS test. The authors, while recognizing the limitations of a static, strength-based test method, welcome comments and suggestions for improvements of this guidance document in future revisions.
**Date**	2017
**URL**	http://dx.doi.org/10.1016/j.dental.2016.11.015
**Extra**	Publisher: The Academy of Dental Materials
**Volume**	33
**Pages**	133–143
**Publication**	Dental Materials
**DOI**	10.1016/j.dental.2016.11.015
**Issue**	2
**Date Added**	1/26/2024, 8:45:09 AM
**Modified**	1/26/2024, 8:45:09 AM

Tags:

Bond strength, Composite, Bonding, Dentin, Enamel, Guidance, Guidelines, Laboratory, Micro-tensile

---

Adhesive application after ceramic surface treatment is detrimental to load-bearing capacity under fatigue of a lithium disilicate glass-ceramic

**Type**	Journal Article
**Author**	Helder Callegaro Velho
**Author**	Lucas Saldanha da Rosa

Author Renatta Wrasse Temp
Author Fernanda Maidana Cocco
Author Gabriel Kalil Rocha Pereira
Author Liliana Gressler May
Author Luiz Felipe Valandro
Date 2022-11
Volume 135
Pages 105453
Publication Journal of the Mechanical Behavior of Biomedical Materials
DOI 10.1016/j.jmbbm.2022.105453
ISSN 17516161
Date Added 1/26/2024, 8:45:04 AM
Modified 1/26/2024, 8:45:04 AM

Aspects of bonding between resin luting cements and glass ceramic materials

Type Journal Article
Author Tian Tian
Author James Kit-Hon Tsoi
Author Jukka P. Matinlinna
Author Michael F. Burrow
Date 2014-07
Volume 30
Pages e147–e162
Publication Dental Materials
DOI 10.1016/j.dental.2014.01.017
Issue 7
ISSN 01095641
Date Added 1/26/2024, 8:45:05 AM
Modified 1/26/2024, 8:45:05 AM

Assessment of Intra-Oral Repair Systems for Veneered Zirconia and Zirconia Only

Type Journal Article
Author Tugçe Merve Ordueri
Author Mehmet Muzaffer Ateş
Author Mutlu Özcan
Abstract <p>The aim of this study was to compare bond strength resin composites to porcelain laminate veneers in the indirect repair method to composite resins used in the direct repair method for cases of porcelain veneer fracture of zirconia-based fixed dental prostheses. In the study, the groups were formed with different percentages of areas to be repaired to mimic porcelain fractures in the mouth. The experimental group of veneered zirconia were as follows: Group A = 100% Zr surface; Group B = 70% Zr, 30% porcelain surface; Group C = 50% Zr, 50% porcelain surface; Group D = 30% Zr, 70% porcelain surface; Group E = 100% porcelain surface. The repairs of the specimens were made using composite resin systems in half of the groups and using porcelain laminate veneers in the other half. Specimens were embedded in acrylic blocks before surface treatments and repairs were applied. After surface conditioning, laminate veneers were applied to the first half of the groups, and composite repair systems were applied to the second half of the groups. After all specimens were aged by thermal cycling, their bond strength values were measured using a Universal Testing Machine, and the obtained data were recorded. The specimens were examined with a stereomicroscope and classified according to failure types (adhesive/cohesive/mixed). Bond strength values were evaluated based on independent-samples

t-test statistics. According to the comparisons among the groups, the bond strength of the indirect repairs made with the laminate material was higher than the bond strength of the repairs made with the composite. There was a statistically significant difference in favor of the indirect repair groups among all groups except for Group C. The highest bond strength was found in Group A in the indirect repair method, while the lowest bond was found in the direct repair method in Group E. Adhesive failure was mostly seen in the groups that were repaired with the composite. </p>

Date 2023-02
Volume 16
Pages 1407
Publication Materials
DOI 10.3390/ma16041407
Issue 4
ISSN 1996-1944
Date Added 1/26/2024, 8:45:06 AM
Modified 1/26/2024, 8:45:06 AM

Attitudes, practice, and experience of German dentists regarding repair restorations

Type Journal Article
Author Philipp Kanzow
Author Robin Hoffmann
Author Claudia Tschammler
Author Jochen Kruppa
Author Tina Rödig
Author Annette Wiegand
Date 2017-05
Volume 21
Pages 1087–1093
Publication Clinical Oral Investigations
DOI 10.1007/s00784-016-1859-3
Issue 4
ISSN 1432-6981
Date Added 1/26/2024, 8:45:03 AM
Modified 1/26/2024, 8:45:03 AM

Bond strength of resin cement to zirconia ceramic with different surface treatments

Type Journal Article
Author Aslıhan Usumez
Author Nermin Hamdemirci
Author Bilge Yuksel Koroglu
Author Irfan Simsek
Author Ozge Parlar
Author Tugrul Sari
Abstract Zirconia-based ceramics offer strong restorations in dentistry, but the adhesive bond strength of resin cements to such ceramics is not optimal. This study evaluated the influence of surface treatments on the bond strength of resin cement to yttrium-stabilized tetragonal zirconia (Y-TZP) ceramic. Seventy-five plates of Y-TZP ceramic were randomly assigned to five groups (n = 15) according to the surface treatments [airborne particle abrasion, neodymium-doped yttrium aluminum garnet (Nd:YAG) laser irradiation (Fidelis Plus 3, Fotona; 2 W, 200 mJ, 10 Hz, with

two different pulse durations 180 or 320 μs), glaze applied, and then 9.5 % hydrofluoric acid gel conditioned, control]. One specimen from each group was randomly selected, and specimens were evaluated with x-ray diffraction and SEM analysis. The resin cement (Clearfil Esthetic Cement, Kuraray) was adhered onto the zirconia surfaces with its corresponding adhesive components. Shear bond strength of each sample was measured using a universal testing machine at a crosshead speed of 1 mm/min. Bond strengths were analyzed through one-way ANOVA/Tukey tests. Surface treatments significantly modified the topography of the Y-TZP ceramic. The Nd:YAG laser-irradiated specimens resulted in both increased surface roughness and bond strength of the resin cement. The highest surface roughness and bond strength values were achieved with short pulse duration. Nd:YAG laser irradiation increased both surface roughness of Y-TZP surfaces and bond strength of resin cement to the zirconia surface. © 2012 Springer-Verlag London Ltd.

Date 2013
URL http://link.springer.com/10.1007/s10103-012-1136-x
Volume 28
Pages 259–266
Publication Lasers in Medical Science
DOI 10.1007/s10103-012-1136-x
Issue 1
Date Added 1/26/2024, 8:45:04 AM
Modified 1/26/2024, 8:45:04 AM

Ceramic materials in dentistry: Historical evolution and current practice

Type Journal Article
Author J R Kelly
Author P Benetti
Abstract Dental ceramics are presented within a simplifying framework allowing for facile understanding of their development, composition and indications. Engineering assessments of clinical function are dealt with and literature is reviewed on the clinical behaviour of all-ceramic systems. Practical aspects are presented regarding the choice and use of dental ceramics to maximize aesthetics and durability, emphasizing what we know and how we know it. © 2011 Australian Dental Association.
Date 2011
Volume 56
Pages 84–96
Publication Australian Dental Journal
DOI 10.1111/j.1834-7819.2010.01299.x
Issue SUPPL. 1
Date Added 1/26/2024, 8:45:04 AM
Modified 1/26/2024, 8:45:04 AM

Tags:

all-ceramic restorations, CAD/CAM, Ceramics, particle-filled glasses, polycrystalline ceramics

---

Cleaning and Surface Treatment Protocols for Repair of Aged Y-TZP with Composite Resin

Type Journal Article
Author Nayara Barchetta
Author Ana Silva

Author Nádia Domingues
Author Sarina Pereira
Author Marco Bottino
Author Guilherme Saavedra
Author Renata de Melo
Author Rodrigo Souza
Date 2021-01
Volume 41
Pages e19–e26
Publication The International Journal of Periodontics & Restorative Dentistry
DOI 10.11607/prd.4915
Issue 1
ISSN 01987569
Date Added 1/26/2024, 8:45:09 AM
Modified 1/26/2024, 8:45:09 AM

Clinical performance of chairside CAD/CAM restorations

Type Journal Article
Author Dennis J. Fasbinder
Date 2006-09
Volume 137
Pages 22S–31S
Publication The Journal of the American Dental Association
DOI 10.14219/jada.archive.2006.0395
ISSN 00028177
Date Added 1/26/2024, 8:45:08 AM
Modified 1/26/2024, 8:45:08 AM

Clinical performance of full rehabilitations with direct composite in severe tooth wear patients: 3.5 Years results

Type Journal Article
Author B A C Loomans
Author C M Kreulen
Author H E C E Huijs-Visser
Author B A M M Sterenborg
Author E M Bronkhorst
Author M C.D.N.J.M. Huysmans
Author N J M Opdam
Abstract Objectives: To evaluate the mid-term clinical performance of direct composite restorations placed in patients with pathological tooth wear needing full rehabilitation with an increase of vertical dimension of occlusion. Methods: In a prospective trial 34 patients (34.0 ± 8.4 years; 25 males, 9 females) were treated with a minimal invasive additive technique using composite restorations. The restorative treatment protocol was to provide all teeth with composite build-up restorations in an increased vertical dimension of occlusion (VDO) using the DSO-technique. Recall appointments were planned after 1 month, 1 and 3 years after treatment. Restorations were scored for clinically acceptability (FDI-criteria) and scores 4 and 5 were recorded as clinically unacceptable. Frequencies of failures and Kaplan Meier survival curves are presented and effect of relevant variables was calculated with a multifactorial Cox regression ($p < 0.05$). Results: 1256 Restorations were placed, 687 anterior, 324 premolar, and 245 molar restorations.

After a mean observation time of 39.7 months a total of 69 failures were observed, of which 61 restorations were repaired (score 4) and 8 were replaced (score 5). Most common reasons for failure were (chip) fractures (n = 43) and caries (n = 11). Placement of anterior restorations in two sessions led to significant 4.6 times more failures then placed in one session. Conclusions: In patients with severe tooth wear a full rehabilitation, in an increased vertical dimension of occlusion, direct composite resin restorations show a 94.8% success and 99.3% survival rate after a period of 3.5 years.

Date 2018

URL https://pubmed.ncbi.nlm.nih.gov/29339203/

Extra Publisher: J Dent

Volume 70

Pages 97–103

Publication Journal of Dentistry

DOI 10.1016/j.jdent.2018.01.001

Date Added 1/26/2024, 8:45:06 AM

Modified 1/26/2024, 8:45:06 AM

Tags:

Clinical study, Composite resin restorations, Increase vertical dimension of occlusion, Interventions, Rehabilitation, Success, Survival, Tooth wear

---

Color Stability of Polymer-Based Composite CAD/CAM Blocks: A Systematic Review

Type Journal Article

Author Gaetano Paolone

Author Mauro Mandurino

Author Francesca De Palma

Author Claudia Mazzitelli

Author Nicola Scotti

Author Lorenzo Breschi

Author Enrico Gherlone

Author Giuseppe Cantatore

Author Alessandro Vichi

Abstract <p>Background: This systematic review aims to evaluate the color stability of resin composite CAD/CAM blocks (CCB) when submitted to staining solutions. Methods: A systematic search was performed on different databases (Embase, Medline, Scopus, Web of Science). Search terms were: ‘polymer infiltrated', ‘polymer-based', ‘resin nanoceramic', ‘resin ceramic', ‘hybrid composite', ‘hybrid ceramic', ‘composite ceramic', ‘resin infiltrated', ‘CAD-CAM', ‘CAD/CAM', ‘color stability', ‘staining', ‘staining susceptibility', ‘color change', ‘color difference'. Inclusion criteria: in vitro articles published in the English language until 18 September 2022 without initial time restriction evaluating the color stability of CCB when submitted to staining solutions. Exclusion criteria: studies investigating color change induced by smoke or whitening treatments; studies not including a clinical evaluation of the results using the thresholds for color perceptibility and acceptability. Risk of bias assessment using the QUIN tool. Findings: Out of the 378 initially retrieved articles, 19 were included in this review. They investigated 17 different CCBs and different artificial staining by liquid protocols, including coffee, red wine, tea, and cola. CCBs exceeded clinical acceptability thresholds for color shift in 18 out of 19 studies, with a significantly higher color stability than conventional hybrid resin-based composites (RBCs), and a significantly lower color stability than ceramic materials. The identified differences in CCBs in color stability can be attributed to the material's composition, but also to the heterogeneity of staining procedures. Interpretation and clinical implication: Clinicians should be aware that, although to a lower degree when compared to RBCs used in direct or indirect procedures, CCBs undergo color changes to a higher degree in comparison to ceramic materials. </p>

Date 2023-01
Volume 15
Pages 464
Publication Polymers
DOI 10.3390/polym15020464
Issue 2
ISSN 2073-4360
Date Added 1/26/2024, 8:45:06 AM
Modified 1/26/2024, 8:45:06 AM

Comparing the Repair of Veneered Zirconia Crowns with Ceramic or Composite Resin: An in Vitro Study

Type Journal Article
Author Hattanas Kumchai
Author Patrapan Juntavee
Author Arthur F. Sun
Author Dan Nathanson
Abstract <p>Statement of problem: Current techniques for repairing porcelain-chipped restorations have several limitations. With advances in CAD/CAM technology, the combination of resin cements and high-strength ceramic materials might offer new options for repairing the chipping of veneering ceramic. Purpose: The purpose of this study is to compare the load-to-failure of veneered zirconia crowns repaired by different materials. Material and Methods: Veneered zirconia crowns were made on aluminum dies (n = 10/group). Feldspathic porcelain (Vita VM9, Vident) was applied to the zirconia coping (Vita In-Ceram YZ, Vident) in a cylindrical shape (Ø 10.5 mm, height 7.5 mm). A bevel cut on the porcelain veneer (45 degree, 3 mm width) was made at one side of each crown to simulate porcelain chipping. The crowns were then divided into four different groups according to the repair materials: 1. Conventional resin composite (A; Tetric EvoCeram, Ivoclar Vivadent); 2. Flowable resin composite (B; G-aenial Universal Flo, GC america); 3. CAD/CAM milled feldspathic ceramic (C; Vita Trilux Forte, Vident); 4. CAD/CAM milled lithium disilicate glass-ceramic (D; IPS e.max CAD, Ivoclar Vivadent). Resin cement (Multilink Automix, Ivoclar Vivadent) was used to cement the CAD/CAM ceramic materials to the beveled crowns. Each crown underwent 5000 cycles of thermocycling. The strength test was performed on an Instron universal testing machine by loading force on the center of repaired part to record load-to-failure. Data were analyzed by ANOVA and Tukey HSD post-hoc tests (α = 0.05). Results: Mean loads-to-failure (in Newton +/− SD) of repaired veneered zirconia crowns were: Gr. A: 660.0 ± 200.5; Gr. B: 681.7 ± 175.9; Gr. C: 1236.0 ± 188.8; Gr. D: 1536.3 ± 286.1. Catastrophic failure was the most dominant failure mode in every group. Few specimens exhibited cohesive failure. Only one specimen in group D had adhesive failure. Conclusions: Within the limitation of the study, veneered zirconia crowns repaired with CAD/CAM ceramic materials have significantly higher load-to-failure than veneered crowns repaired with resin composite (p ≤ 0.05). Clinical Implications: Traditionally, porcelain-chipped restorations are often repaired with resin composite and bonding technique. Repairing chipped porcelain with CAD/CAM ceramics fitting the fractured parts can be alternative option with potential advantages. More well-designed studies are necessary to justify this novel repair technique.</p>
Date 2020-04
Volume 8
Pages 37
Publication Dentistry Journal
DOI 10.3390/dj8020037
Issue 2
ISSN 2304-6767
Date Added 1/26/2024, 8:45:06 AM
Modified 1/26/2024, 8:45:06 AM

Comparison of endocrowns made of lithium disilicate glass-ceramic or polymer-infiltrated ceramic networks and direct composite resin restorations: fatigue performance and stress distribution

**Type**	Journal Article
**Author**	Gustavo Dartora
**Author**	Gabriel Kalil Rocha Pereira
**Author**	Rodrigo Varella de Carvalho
**Author**	Camila Pauleski Zucuni
**Author**	Luiz Felipe Valandro
**Author**	Paulo Francisco Cesar
**Author**	Ricardo Armini Caldas
**Author**	Atais Bacchi
**Date**	2019-12
**Volume**	100
**Pages**	103401
**Publication**	Journal of the Mechanical Behavior of Biomedical Materials
**DOI**	10.1016/j.jmbbm.2019.103401
**ISSN**	17516161
**Date Added**	1/26/2024, 8:45:08 AM
**Modified**	1/26/2024, 8:45:08 AM

Comparison of traditional and simplified methods for repairing CAD/CAM feldspathic ceramics

**Type**	Journal Article
**Author**	Michele Carrabba
**Author**	Alessandro Vichi
**Author**	Chris Louca
**Author**	Marco Ferrari
**Date**	2017
**Volume**	9
**Pages**	257
**Publication**	The Journal of Advanced Prosthodontics
**DOI**	10.4047/jap.2017.9.4.257
**Issue**	4
**ISSN**	2005-7806
**Date Added**	1/26/2024, 8:45:08 AM
**Modified**	1/26/2024, 8:45:08 AM

Current status on lithium disilicate and zirconia: a narrative review

**Type**	Journal Article
**Author**	Fernando Zarone
**Author**	Maria Irene Di Mauro
**Author**	Pietro Ausiello
**Author**	Gennaro Ruggiero
**Author**	Roberto Sorrentino

Date 2019-12
Volume 19
Pages 134
Publication BMC Oral Health
DOI 10.1186/s12903-019-0838-x
Issue 1
ISSN 1472-6831
Date Added 1/26/2024, 8:45:04 AM
Modified 1/26/2024, 8:45:04 AM

Defective restoration repair or replacement

Type Journal Article
Author Juliana B. da Costa
Author Kevin Frazier
Author Mai-Ly Duong
Author Sharukh Khajotia
Author Purnima Kumar
Author Olivia Urquhart
Date 2021-04
Volume 152
Pages 329–330.e2
Publication The Journal of the American Dental Association
DOI 10.1016/j.adaj.2021.01.011
Issue 4
ISSN 00028177
Date Added 1/26/2024, 8:45:08 AM
Modified 1/26/2024, 8:45:08 AM

Dental ceramics

Type Journal Article
Author J R Kelly
Date 2008
URL http://dx.doi.org/10.14219/jada.archive.2008.0359
Extra Publisher: Â© 2008 American Dental Association
Volume 207
Pages 89
Publication British Dental Journal
DOI 10.1038/sj.bdj.2009.652
Issue 2
Date Added 1/26/2024, 8:45:07 AM
Modified 1/26/2024, 8:45:07 AM

Dental Ceramics for Restoration and Metal Veneering

Type Journal Article
Author Yu Zhang

Author J. Robert Kelly
Date 2017-10
Volume 61
Pages 797–819
Publication Dental Clinics of North America
DOI 10.1016/j.cden.2017.06.005
Issue 4
ISSN 00118532
Date Added 1/26/2024, 8:45:04 AM
Modified 1/26/2024, 8:45:04 AM

Effect of active application of self-etching ceramic primer on the long-term bond strength of different dental CAD/CAM materials

Type Journal Article
Author J. Tribst
Author P. Diamantino
Author M. de Freitas
Author I. Tanaka
Author L. Silva-Concílio
Author R. de Melo
Author GS. Saavedra
Date 2021
URL http://www.medicinaoral.com/medoralfree01/aop/58723.pdf
Pages e1089–e1095
Publication Journal of Clinical and Experimental Dentistry
DOI 10.4317/jced.58723
ISSN 19895488
Date Added 1/26/2024, 8:45:04 AM
Modified 1/26/2024, 8:45:04 AM

Effect of chemical etching solutions versus air abrasion on the adhesion of self-adhesive resin cement to IPS e.max ZirCAD with and without aging.

Type Journal Article
Author Amal Abdelsamad Sakrana
Author Mutlu Özcan
Abstract OBJECTIVES: This study evaluated the adhesion of self-adhesive resin cement to zirconia after various surface conditioning methods in non-aged and aged conditions. METHODS: Zirconia (IPS e.max ZirCAD, Ivoclar Vivadent) and resin composite blocks were obtained and randomly divided into four groups according to one of the following surface conditioning methods: C: No surface treatment (Control); AS: Air abrasion (50 µm Al_2O_3) at low pressure (0.2 MPa) plus silane (Monobond Plus, Ivoclar Vivadent); MC: Methylene chloride for 60 min; CE: Chemical etching solution at 100°C for 60 min. Surface roughness (Ra) measurements were performed on the control and conditioned zirconia specimens using a profilometer. Each conditioned zirconia block was bonded to its corresponding resin composite block using self-adhesive resin cement (RelyX Unicem Aplicap, 3M ESPE) under a load of 300 g. Half of the groups were tested without aging; the other half were tested after x6000 thermocycling. Sticks were obtained from bonded specimens and subjected to microtensile bond strength (MTBS) testing in a universal testing machine (0.5 mm/min). Failure types were classified after debonding. Data (MPa) were analyzed using two-way ANOVA and Tukey's tests ($\alpha = 0.05$). RESULTS: The highest mean Ra

value was obtained in Group CE (0.52 μm), and the lowest in Group C (0.25 μm). Significantly lower MTBS test results were obtained in Group C, both in non-aged (17.4 ± 1.1 MPa) and aged (5.11 ± 0.5 MPa) conditions than in the other groups (34.6 ± 1.5 to 51.2 ± 1.1 MPa; 28.0 ± 0.9 to 49.5 ± 1.0 MPa in non-aged and aged conditions, respectively) (P < 0.05). Group CE presented the highest MTBS test results in both non-aged and aged conditions (51.2 ± 1.1 MPa and 49.5 ± 1 MPa, respectively) (P < 0.05). Failure types were predominantly adhesive in all groups before and after aging. After aging, less adhesive and more mixed and cohesive failures in cement were observed in Groups AS and CE than in the other groups. CONCLUSION: Chemical etching of zirconia may have potential use as a substitute for air abrasion as a surface conditioning method for zirconia.

Date 2017

Volume 12

Pages 72–85

Publication The international journal of esthetic dentistry

Issue 1

Date Added 1/26/2024, 8:45:05 AM

Modified 1/26/2024, 8:45:05 AM

Tags:

chemistry, Dental, Resin Cements, Surface Properties, Adhesives, Air Abrasion, Solutions

---

Effect of different repair methods on the bond strength of resin composite to CAD/CAM materials and microorganisms adhesion: An in situ study

Type Journal Article

Author Aretha Heitor Veríssimo

Author Dayanne Monielle Duarte Moura

Author Amanda Maria de Oliveira Dal Piva

Author Marco Antonio Bottino

Author Leopoldina de Fátima Dantas de Almeida

Author Adriana da Fonte Porto Carreiro

Author Rodrigo Othávio de Assunção e Souza

Abstract Objective: To evaluate the influence of different repair protocols andin situ aging on colony forming units (CFU) and shear bond strength (SBS) between CAD/CAM materials and resin composite. Methodology: 150 blocks (6 × 5 × 2.5 mm) were made out of each restorative material (CAD/CAM resin composite -RC), polymer-infiltrated ceramic - PIC and glass ceramic (VS), totaling 450 blocks. Fifty blocks of each material were submitted to a 60-day in situ aging by fixing the blocks into cavities prepared in the posterior region of the base of complete dentures. The aged and non-aged blocks were randomly divided into 30 groups of 10 (N = 300) according to the following factors: “Restorative material”, “Surface Treatment”, and “Aging”. Z350 resin composite cylinders were made on the surface of the blocks, and the specimens were submitted to thermocycling, shear test, failure mode analysis, and complementary analyses of roughness, fungal and bacterial CFUs, SEM, and EDS. The SBS (MPa) and CFU/mL data were statistically analyzed by ANOVA and Tukey's test (5%). The other variables were analyzed by qualitative analyzes. Results: The “aging” factor was significant for RC and VS and the “surface treatment” factor was significant for the three restorative materials. Hydrofluoric (HF) acid etching followed by silanization was the best protocol for PIC and VS and diamond bur + SBU was the best protocol for RC. CFU/mL was similar among the restorative materials. Conclusion: In situ aging reduced the bond strength between the resin composite repair and RC and VS materials. Clinical Significance: The repair protocol for each restorative material is different and may be influenced by the time of clinical use.

Date 2020-02

Extra Publisher: Elsevier Ltd PMID: 31863809

Volume 93

Publication Journal of Dentistry
DOI 10.1016/j.jdent.2019.103266
ISSN 03005712
Date Added 1/26/2024, 8:45:03 AM
Modified 1/26/2024, 8:45:03 AM

Tags:

Bond strength, CAD / CAM, Dental ceramics, Microbiology, Shear strength, Surface treatment

---

Effect of Different Surface Treatments on Repair Bond Strength of CAD/CAM Resin-Matrix Ceramics

Type Journal Article
Author Semih Arkoy
Author Mutahhar Ulusoy
Abstract <p>The purpose of this study was to investigate the influence of different surface treatment methods on the micro-tensile bond strength (µTBS) of resin-matrix ceramic (RMC) blocks repaired with resin composite. Three different prefabricated RMC blocks including Lava Ultimate (LU), Grandio Blocs (GB), and Shofu Block HC (HC) were thermo-cycled and divided into five surface treatment groups: Control (C), bur grinding (G), airborne particle abrasion (APA), Er,Cr:YSGG laser irritation (LI), and APA combined with LI (APA+LI). After surface treatments, topographic alterations were examined by scanning electron microscopy. Then, Universal Adhesive (Single Bond Universal) was applied and repair was simulated with nanohybrid composite (Grandio SO). Bonded specimens were cut into 1 mm2 sized beams (n = 16) and a µTBS test was conducted by using a universal test machine. Fracture types were evaluated by using a stereomicroscope. The bond- strength data was evaluated by two-way ANOVA and Tukey post-hoc test ($\alpha = 0.05$). The µTBS values were significantly affected by the surface treatment variable and the interaction terms of the variables ($p \leq 0.001$). However, no significant effect of RMC type was detected ($p > 0.05$). Among all materials, GBAPA+LI indicated the highest µTBS value. Except for the GBC, all surface treatments showed clinically acceptable bond-strength values. However, the surface treatments applied to GB and LU before the repair processes increased the repair bond-strength values while causing a negative effect for HC. In addition, LI and APA+LI can be applied as an alternative route compared to other procedures recommended by the manufacturer for surface preparation in intraoral RMC repair. </p>
Date 2022-09
Volume 15
Pages 6314
Publication Materials
DOI 10.3390/ma15186314
Issue 18
ISSN 1996-1944
Date Added 1/26/2024, 8:45:09 AM
Modified 1/26/2024, 8:45:09 AM

Effect of Different Universal Bonding Agent Procedures on Repair of Feldspathic and Hybrid Ceramics

Type Journal Article
Author Mustafa Atala
Author Elif Yeğin

Abstract PURPOSE: To perform application of universal bonding agent based only on the manufacturer's instructions for CAD/CAM-manufactured feldspathic and hybrid ceramics and to investigate the bond strength of the composite resin to these ceramics. MATERIALS AND METHODS: A total of 120 samples (2-mm thickness) were obtained from three different CAD/CAM-manufactured ceramics each: a feldspathic ceramic (Vitablocs Mark II), a hybrid ceramic (Cerasmart, GC), and a dual-network ceramic (Vita Enamic). Each of these restorative materials was divided into six subgroups (n = 20 each) in order to apply universal adhesives: All-Bond Universal, Clearfil Quick Universal, Premio Bond, Optibond XTR, Prime&Bond, and Tokuyama Universal Bond. Each bonding agent was applied according to the manufacturer's instructions. Microshear bond strength test was performed at a crosshead speed of 1 mm/min. Two-way analysis of variance (ANOVA) and Tukey post hoc tests were used for statistical analyses. RESULTS: The data suggested a significant impact of bonding agent on all restorative materials applied ($P < .001$); however, only Prime&Bond demonstrated different effects between materials, with a significant difference between Vitablocs Mark II and Cerasmart restorative materials ($P = .001$). CONCLUSION: All universal bonding agents provided an acceptable bond strength for each ceramic. Since different universal bonding agents affected the bond strength between ceramics and composite resin, selecting the most suitable bonding agent for each material will increase the bond strength and clinical success.

Date 2022-05
Extra Publisher: Quintessence Publishing PMID: 35727264
Volume 35
Pages 330–337
Publication The International Journal of Prosthodontics
DOI 10.11607/ijp.7753
Issue 3
ISSN 08932174
Date Added 1/26/2024, 8:45:09 AM
Modified 1/26/2024, 8:45:09 AM

Effect of Finishing Time on Microleakage at the Composite-Repair Interface

Type Journal Article
Author Fereshteh Shafiei
Author Nazanin Berahman
Author Elmira Niazi
Date 2016-09
Volume 10
Pages 497–504
Publication The Open Dentistry Journal
DOI 10.2174/1874210601610010497
Issue 1
ISSN 1874-2106
Date Added 1/26/2024, 8:45:05 AM
Modified 1/26/2024, 8:45:05 AM

Effect of finishing/polishing techniques and aging on topography, C. albicans adherence, and flexural strength of ultra-translucent zirconia: an in situ study

Type Journal Article
Author Isabelle Helena Gurgel de Carvalho
Author Nathalia Ramos da Silva
Author Taciana Emília Leite Vila-Nova

**Author**	Leopoldina de Fatima Dantas de Almeida
**Author**	Aretha Heitor Veríssimo
**Author**	Renata Marques de Melo
**Author**	Yu Zhang
**Author**	Rodrigo Othávio de Assunção e Souza
**Date**	2022
**Extra**	Publisher: Clin Oral Investig
**Volume**	26
**Pages**	889–900
**Publication**	Clinical Oral Investigations
**DOI**	10.1007/s00784-021-04068-3
**Issue**	1
**Date Added**	1/26/2024, 8:45:04 AM
**Modified**	1/26/2024, 8:45:04 AM

Tags:

doi:10.1007/s00784-021-04068-3, Isabelle Helena Gurgel de Carvalho, MEDLINE, Nathalia Ramos da Silva, National Center for Biotechnology Information, National Institutes of Health, National Library of Medicine, NCBI, NIH, NLM, pmid:34448074, PubMed Abstract, Rodrigo Othávio de Assunção E Souza

---

Effect of hydrofluoric acid concentration and etching duration on select surface roughness parameters for zirconia

**Type**	Journal Article
**Author**	Beata Smielak
**Author**	Leszek Klimek
**Date**	2015-06
**Volume**	113
**Pages**	596–602
**Publication**	The Journal of Prosthetic Dentistry
**DOI**	10.1016/j.prosdent.2015.01.001
**Issue**	6
**ISSN**	00223913
**Date Added**	1/26/2024, 8:45:05 AM
**Modified**	1/26/2024, 8:45:05 AM

Effect of multiple firings on surface roughness and flexural strength of CAD-CAM ceramics

**Type**	Journal Article
**Author**	Meryem Gülce Subaşı
**Author**	Gülce Çakmak
**Author**	Murat Sert
**Author**	Burak Yilmaz
**Date**	2022-08
**Volume**	128
**Pages**	216.e1–216.e8
**Publication**	The Journal of Prosthetic Dentistry

DOI 10.1016/j.prosdent.2022.05.021
Issue 2
ISSN 00223913
Date Added 1/26/2024, 8:45:05 AM
Modified 1/26/2024, 8:45:05 AM

Effect of primer treatment on bonding of resin cements to zirconia ceramic.

Type Journal Article

Author Shuzo Kitayama
Author Toru Nikaido
Author Rena Takahashi
Author Lei Zhu
Author Masaomi Ikeda
Author Richard M Foxton
Author Alireza Sadr
Author Junji Tagami

Abstract OBJECTIVES: To evaluate and compare bond strengths of different primers and resin cements to silica-based and zirconia ceramics. METHODS: Silica-based and zirconia ceramic specimens were ground flat with #600-grit SiC paper. The ceramic surfaces were airborne-particle abraded and then divided into 11 groups of seven each: untreated (control); and conditioned with one of the six primers in combination with a resin cement from the same manufacturer as follows: Bistite II/Tokuso Ceramic Primer, Linkmax/GC Ceramic Primer, RelyX ARC/RelyX Ceramic Primer, Panavia F 2.0/Clearfil Ceramic Primer, and Resicem/Shofu Porcelain Primer and Resicem/AZ Primer. Stainless steel rods were bonded to the ceramic surfaces using one of the five resin cements. After 24-h water storage, the tensile bond strengths were tested using a universal testing machine and failure modes were examined. RESULTS: Conditioning with primers containing a silane coupling agent (all the primers except AZ Primer) significantly enhanced bond strengths of resin cements to silica-based ceramic. For zirconia ceramic, Resicem/AZ Primer exhibited significantly higher bond strength than the other groups except Panavia F 2.0/Clearfil Ceramic Primer. The predominant failure mode of the groups conditioned with primers containing a phosphonic acid monomer (AZ Primer) or a phosphate ester monomer (Clearfil Ceramic Primer and Tokuso Ceramic Primer) was cohesive failure in cements whereas that with the other primers was adhesive failure at the zirconia surfaces. SIGNIFICANCE: The use of primers containing a silane coupling agent improved resin bonding to silica-based ceramic. On the other hand, the use of primers containing a phosphonic acid monomer or a phosphate ester monomer improved resin bonding to zirconia ceramic.

Date 2010
Volume 26
Pages 426–432
Publication Dental materials : official publication of the Academy of Dental Materials
DOI 10.1016/j.dental.2009.11.159
Issue 5
Date Added 1/26/2024, 8:45:07 AM
Modified 1/26/2024, 8:45:07 AM

Tags:

Dental Bonding, Dental Porcelain, Dental Stress Analysis, Materials Testing, methods, Resin Cements, Methacrylates, Silanes, Yttrium, Zirconium, analogs & derivatives, Analysis of Variance, Nonparametric, Organophosphonates, Phosphonoacetic Acid, Silicon Dioxide, Statistics, Tensile Strength

Effect of Surface Preparation with Nd:YAG and Er,Cr:YSGG Lasers on the Repair Bond Strength of Lithium Disilicate Glass Ceramic to a Silorane-based Composite Resin

Type Journal Article
Author Mohammad Esmaeel Ebrahimi Chaharom
Author Fatemeh Pournaghi Azar
Author Narmin Mohammadi
Author Rezvan Nasiri
Abstract Background. This study was undertaken to evaluate the repair bond strength of lithium disilicate glass ceramic to a silorane-based composite resin after surface preparation with Nd:YAG and Er,Cr:YSGG lasers. Methods. A total of 102 lithium disilicate glass ceramic samples (IPS e.max Press), measuring 5 mm in diameter and 4 mm in thickness, were randomly assigned to 6 groups (n=17): group 1, no surface preparation (control); group 2, acid etching with 9.5% hydrofluoric acid (HF); group 3, surface preparation with 4.5-W Nd:YAG laser; group 4, surface preparation with 6-W Nd:YAG laser; group 5, surface preparation with 1.5-W Er,Cr:YSGG laser; and group 6, surface preparation with 6-W Er,Cr:YSGG laser. After preparation of surfaces and application of silane, all the samples were repaired with the use of a silorane-based composite resin, followed by storage in distilled water at a temperature of 37°C for 24 hours and thermocycling. Finally, the samples were subjected to a shearing bond strength test; the fracture modes were determined under a stereomi-croscope. Results. There were significant differences between the HF group and the other groups (P=0.000). Two-by-two comparisons of the other groups revealed no significant differences (P>0.05). Conclusion. Use of HF proved the most effective surface preparation technique to increase the repair bond strength between lithium disilicate glass ceramic and silorane-based composite resin; compared to the control group.
Date 2018-03
URL http://joddd.tbzmed.ac.ir/Abstract/joddd-16826
Extra Publisher: Maad Rayan Publishing Company
Volume 12
Pages 12–17
Publication Journal of Dental Research, Dental Clinics, Dental Prospects
DOI 10.15171/joddd.2018.003
Issue 1
ISSN 2008-210X
Date Added 1/26/2024, 8:45:08 AM
Modified 1/26/2024, 8:45:08 AM

Effect of surface pretreatments on the microtensile bond strength of lithium-disilicate ceramic repaired with composite resin

Type Journal Article
Author Regina Cláudia Ramos Colares
Author Jiovanne Rabelo Neri
Author André Mattos Brito de Souza
Author Karina Matthes de Freitas Pontes
Author Juliano Sartori Mendonça
Author Sérgio Lima Santiago
Abstract The aim of this study was to evaluate the influence of ceramic surface treatments and silane drying temperature on the microtensile bond strength (μTBS) of a resin composite to a lithium disilicate ceramic. Twenty blocks (7x7x5 mm) of lithium disilicate-based hotpressed ceramic were fabricated and randomly divided into 4 groups: G1: acid etching with 9.5% hydrofluoric acid for 20 s and drying silane with room-temperature air; G2: acid etching with 9.5% hydrofluoric acid for 20 s and drying silane with 45 ± 5 °C warm air; G3: airborne-particle abrasion with 50 μm aluminum oxide particles and drying silane with 45 ± 5 °C warm air; G4:

airborne-particle abrasion with 50 µm aluminum oxide particles and drying silane with air at room-temperature. After treatments, an adhesive system (Single Bond 2) was applied, light-cured and direct restorations were built up with a resin composite (Filtek Z250). Each specimen was stored in distilled water at 37 °C for 24 h and cut into ceramic-composite beams with 1 mm2 of cross-sectional area for µTBS testing. Statistical analysis was performed with one-way ANOVA and Student- Newman-Keuls test (α=0.05). µTBS means (S.D.) in MPa were: G1: 32.14 (7.98), G2: 35.00 (7.77) and G3: 18.36 (6.17). All specimens of G4 failed during the cutting. G1 and G2 presented significantly higher µTBS than G3 (p<0.05). There was no statistically significant difference between G1 and G2 (p>0.05). As far as the bond strength is concerned, surface pretreatment of lithium-disilicate ceramic with hydrofluoric acid and silane application can be used as an alternative to repair ceramic restorations with composite resin, while surface pretreatment with sandblasting should be avoided.

**Date**	2013-07
**Extra**	PMID: 24173254
**Volume**	24
**Pages**	349–352
**Publication**	Brazilian Dental Journal
**DOI**	10.1590/0103-6440201301960
**Issue**	4
**ISSN**	01036440
**Date Added**	1/26/2024, 8:45:08 AM
**Modified**	1/26/2024, 8:45:08 AM

Tags:

Bond strength, Surface treatment, Lithium disilicate ceramic

Effect of Surface Roughness on Stain Resistance of Dental Resin Composites

**Type**	Journal Article
**Author**	Huan Lu
**Author**	Leslie B. Roeder
**Author**	Lei Lei
**Author**	John M. Powers
**Date**	2005-03
**URL**	https://onlinelibrary.wiley.com/doi/10.1111/j.1708-8240.2005.tb00094.x
**Volume**	17
**Pages**	102–108
**Publication**	Journal of Esthetic and Restorative Dentistry
**DOI**	10.1111/j.1708-8240.2005.tb00094.x
**Issue**	2
**ISSN**	1496-4155
**Date Added**	1/26/2024, 8:45:06 AM
**Modified**	1/26/2024, 8:45:06 AM

Effect of surface treatments and universal adhesive application on the microshear bond strength of CAD/CAM materials

**Type**	Journal Article
**Author**	Soner Şişmanoğlu
**Author**	Aliye Tuğçe Gürcan

Author Zuhal Yıldırım-Bilmez
Author Rana Turunç-Oğuzman
Author Burak Gümüştaş
Date 2020
Volume 12
Pages 22
Publication The Journal of Advanced Prosthodontics
DOI 10.4047/jap.2020.12.1.22
Issue 1
ISSN 2005-7806
Date Added 1/26/2024, 8:45:04 AM
Modified 1/26/2024, 8:45:04 AM

Effect of surface treatments on shear bond strength of resin composite bonded to CAD/CAM resin-ceramic hybrid materials

Type Journal Article
Author Merve Bankoğlu Güngör
Author Seçil Karakoca Nemli
Author Bilge Turhan Bal
Author Senem Ünver
Author Aylin Doğan
Date 2016
Volume 8
Pages 259
Publication The Journal of Advanced Prosthodontics
DOI 10.4047/jap.2016.8.4.259
Issue 4
ISSN 2005-7806
Date Added 1/26/2024, 8:45:07 AM
Modified 1/26/2024, 8:45:07 AM

Effect of various surface conditioning methods on the adhesion of dual-cure resin cement with MDP functional monomer to zirconia after thermal aging.

Type Journal Article
Author Mutlu Ozcan
Author Henk Nijhuis
Author Luiz Felipe Valandro
Abstract This study evaluated the effect of chairside and laboratory types of surface conditioning methods on the adhesion of dual-cure resin cement with MDP functional monomer to zirconia ceramic after thermocycling. Disk-shaped (diameter: 10 mm, thickness: 2 mm) Y-TZP ceramics (Lava, 3M ESPE) were used (N=40) and finished with wet 1200-grit silicon carbide abrasive paper. Specimens were randomly divided into four experimental groups according to the following surface conditioning methods (n=10 per group): Group 1–Chairside airborne particle abrasion with 50-microm Al2O2 + Alloy Primer (Kuraray); Group 2–Airborne particle abrasion with 50-microm Al2O3 + Cesead II Opaque Primer (Kuraray); Group 3–Airborne particle abrasion with 50-microm A12O3 + Silano-Pen + silane coupling agent (Bredent); Group 4–Laboratory tribochemical silica coating (110-microm Al2O3 + 110-microm SiOx) (Rocatec) + silane coupling agent (ESPE-Sil). Adhesive cement, Panavia F 2.0 (Kuraray), was bonded incrementally to the ceramic surfaces using polyethylene molds (diameter: 3.6 mm, height: 5

mm). All specimens were thermocycled (5 and 55 degrees C, 6,000 cycles) and subjected to shear bond strength test (1 mm/min). Data were statistically analyzed (one-way ANOVA, alpha=0.05), whereby no significant differences were found among the four groups (8.43+/-1.3, 8.98+/-3.6, 12.02+/-6.7, and 8.23+/-3.8 MPa) (p=0.1357). Therefore, the performance of chairside conditioning methods used for zirconia was on par with the laboratory alternative tested.

Date 2008
Volume 27
Pages 99–104
Publication Dental materials journal
Issue 1
Date Added 1/26/2024, 8:45:06 AM
Modified 1/26/2024, 8:45:06 AM

Tags:

chemistry, Dental Bonding, Dental Porcelain, Materials Testing, Resin Cements, Surface Properties, Aluminum Oxide, Carbon Compounds, Dental Cements, Humans, Inorganic, Mechanical, Methacrylates, Organophosphorus Compounds, Shear Strength, Silanes, Silicon Compounds, Stress, Temperature, Thiones, Time Factors, Yttrium, Zirconium

---

Effects of different surface treatments on ceramic repairs with composite.

Type Journal Article
Author Bi Rong Huang
Author Xiao Yan Wang
Author Xue Jun Gao
Abstract To compare different surface treatments of lithium disilicate-based ceramic on bond strengths to composite. Lithium disilicate-based ceramic microbars (1 mm × 1 mm × 6 mm) were produced and abraded using diamond (D) or CoJet sandblasting (C), etched using phosphoric acid (P) or hydrofluoric acid (H) and silanised (S). A total of 12 surface treatments were adopted: D, DP, DH, DS, DPS, DHS, C, CP, CH, CS, CPS and CHS. An adhesive resin (XP Bond) was then applied and a composite build-up was placed. All specimens were thermocycled 5,000 times and submitted to microtensile bond strength test (μTBS). Results were analysed using one-way analysis of variance and the Dunnett T3 test. Stereomicroscope and scanning electron microscopy were used to determine the failure modes. Surface roughening, acid etching and silanisation all have a significant influence on the ceramic-composite bond durability. Groups DH (30.7 ± 6.5 MPa), DHS (32.0 ± 8.2 MPa), CH (27.2 ± 4.5 MPa) and CHS (29.2 ± 5.5 MPa) showed the highest μTBS statistically. Groups DS (11.5 ± 3.0 MPa) and DPS (9.7 ± 2.2 MPa) had higher μTBS than groups D (4.5 ± 2.9 MPa) and DP (5.2 ± 3.0 MPa), while groups C (1.7 ± 0.7 MPa), CS (1.7 ± 0.9 MPa), CP (1.7 ± 0.6 MPa) and CPS (1.7 ± 1.1 MPa) showed the lowest μTBS. Hydrofluoric acid etching was effective in improving the bond strength of composite to lithium disilicate-based ceramic, regardless of the method of surface roughening and silanisation.
Date 2013
Volume 16
Pages 111–117
Publication The Chinese journal of dental research : the official journal of the Scientific Section of the Chinese Stomatological Association (CSA)
Issue 2
Date Added 1/26/2024, 8:45:07 AM
Modified 1/26/2024, 8:45:07 AM

Tags:

Ceramics, Electron, Microscopy, Scanning, Surface Properties, Tensile Strength, ceramic repair, Composite Resins, Dental Materials, diamond, hydrofluoric acid, microtensile, silane

---

Effects of surface treatments on repair bond strength of a new CAD/CAM ZLS glass ceramic and two different types of CAD/CAM ceramics

**Type**	Journal Article
**Author**	Ayse Seda Ataol
**Author**	Gulfem Ergun
**Abstract**	We evaluated the effects of three different surface treatments and two different bonding procedures on shear bond strength of resin composites to different computer-aided design/manufacturing (CAD/CAM) ceramics with or without thermocycling. The test specimens were prepared from three different CAD/CAM ceramics (Group A, IPS e.max CAD; Group B, IPS e.max ZirCAD; Group C, Vita Suprinity). They were divided into eight subgroups according to the surface treatment and bonding procedures. After resin composite was applied to the surfaces of all test specimens, they were stored in distilled water for 24 h at 37°C. Next, half of the test specimens were subjected to a thermocycling procedure, and the other half were stored in distilled water at 37°C throughout thermocycling. The shear bond strength was performed using a universal testing machine. Statistical analysis revealed that the ceramic types ($P > 0.0031$) and thermocycling ($P > 0.0021$) did not, but the surface treatment technique did significantly affect the shear bond strength values ($P < 0.05$; except group C). Higher bond strength values were observed with HF etching in Groups A and C compared with the other tested surface treatments.
**Date**	2018-06
**Extra**	Publisher: Nihon University, School of Dentistry PMID: 29925704
**Volume**	60
**Pages**	201–211
**Publication**	Journal of Oral Science
**DOI**	10.2334/josnusd.17-0109
**Issue**	2
**ISSN**	18804926
**Date Added**	1/26/2024, 8:45:09 AM
**Modified**	1/26/2024, 8:45:09 AM

Tags:

Repair bond strength, Bonding agents, CAD/CAM ceramics, Resin composite, Surface treatments

---

Evaluating Ceramic Repair Materials in Terms of Bond Strength and Color Stability

**Type**	Journal Article
**Author**	Fatma Sanal
**Author**	Hamiyet Kilinc
**Abstract**	Purpose: To evaluate the shear bond strength (SBS) and color stability (ΔE) of four ceramic veneers (VITA VM 9; VITA VM 13; VITA VMK 95; and IPS e.max Ceram) following repair with three different self-adhesive composite resins (Fusio Liquid Dentin; Constic; and Vertise Flow) and BISCO Intraoral Repair Kit + Filtek Supreme (BC + FS). Materials and Methods: A total of 96 specimens were prepared per ceramic material, and each ceramic group was divided into subgroups according to testing method (32 specimens for color stability analysis and 64 specimens for SBS testing). ΔE and SBS (SBS1 = non-aged, SBS2 = thermally aged) values of the repaired specimens were calculated. One specimen from each of the thermally aged groups

was investigated under scanning electron microscopy (SEM). Two-way ANOVA was used to analyze the mean ΔE, SBS1, and SBS2 values. The differences between SBS1 and SBS2 values were analyzed using independent-samples t test. Results: The lowest ΔE values were calculated for Constic ($P \le .05$). The SBS2 values among all groups were lower than the SBS1 values ($P \le .05$). The lowest SBS2 values were observed for Vertise Flow (2.3 ± 1.47 MPa) ($P \le .05$) and were below the acceptable limit of 5 MPa. Conclusion: All of the investigated repair materials in combination with the ceramic veneers exhibited ΔE values that were higher than the clinically acceptable limits. Thermal aging negatively affected the SBS values in all groups ($P \le .05$). All tested self-adhesive composite resins, with the exception of Vertise Flow, could be used instead of a repair kit with flowable composite resin, especially in noncooperative patients, for the purpose of repairing chipping fractures for VITA VM 9, VITA VM 13, and VITA VMK 95 ($P > .05$). Int J Prosthodont 2020;33:536-545. doi: 10.11607/ijp.6760 © 2020. by Quintessence Publishing Co Inc.

Date 2020-09
Extra Publisher: Quintessence Publishing PMID: 32956435
Volume 33
Pages 536–545
Publication The International Journal of Prosthodontics
DOI 10.11607/ijp.6760
Issue 5
ISSN 08932174
Date Added 1/26/2024, 8:45:04 AM
Modified 1/26/2024, 8:45:04 AM

Evaluation of shear bond strength of zirconia-based monolithic CAD-CAM materials to resin cement after different surface treatments

Type Journal Article
Author B Altan
Author S Cinar
Author B Tuncelli
Date 2019
Volume 22
Pages 1475
Publication Nigerian Journal of Clinical Practice
DOI 10.4103/njcp.njcp_157_19
Issue 11
ISSN 1119-3077
Date Added 1/26/2024, 8:45:10 AM
Modified 1/26/2024, 8:45:10 AM

Evaluation of the repair capacities and color stabilities of a resin nanoceramic and hybrid CAD/CAM blocks

Type Journal Article
Author Hasibe Sevilay Bahadır
Author Yusuf Bayraktar
Date 2020
Volume 12
Pages 140
Publication The Journal of Advanced Prosthodontics

**DOI**	10.4047/jap.2020.12.3.140
**Issue**	3
**ISSN**	2005-7806
**Date Added**	1/26/2024, 8:45:09 AM
**Modified**	1/26/2024, 8:45:09 AM

Extending the service life of existing dental restorations with esthetic and functional limitations

**Type**	Journal Article
**Author**	Malin Strasding
**Author**	Vincent Fehmer
**Author**	Bjarni E. Pjetursson
**Author**	Irena Sailer
**Date**	2018-06
**Volume**	119
**Pages**	893–896
**Publication**	The Journal of Prosthetic Dentistry
**DOI**	10.1016/j.prosdent.2017.08.011
**Issue**	6
**ISSN**	00223913
**Date Added**	1/26/2024, 8:45:05 AM
**Modified**	1/26/2024, 8:45:05 AM

FDI policy statement on Minimal Intervention Dentistry (MID) for managing dental caries

**Type**	Journal Article
**Author**	FDI World Dental Federation
**Date**	2017-02
**Volume**	67
**Pages**	6–7
**Publication**	International Dental Journal
**DOI**	10.1111/idj.12308
**Issue**	1
**ISSN**	00206539
**Date Added**	1/26/2024, 8:45:08 AM
**Modified**	1/26/2024, 8:45:08 AM

FDI World Dental Federation: clinical criteria for the evaluation of direct and indirect restorations—update and clinical examples

**Type**	Journal Article
**Author**	Reinhard Hickel
**Author**	Arnd Peschke
**Author**	Martin Tyas
**Author**	Ivar Mjör

Author Stephen Bayne
Author Mathilde Peters
Author Karl-Anton Hiller
Author Ross Randall
Author Guido Vanherle
Author Siegward D. Heintze
Date 2010-08
Volume 14
Pages 349–366
Publication Clinical Oral Investigations
DOI 10.1007/s00784-010-0432-8
Issue 4
ISSN 1432-6981
Date Added 1/26/2024, 8:45:07 AM
Modified 1/26/2024, 8:45:07 AM

Fractography of clinical failures of indirect resin composite endocrown and overlay restorations

Type Journal Article
Author Carlo M. Saratti
Author Giovanni T. Rocca
Author Stéphane Durual
Author Ulrich Lohbauer
Author Jack L. Ferracane
Author Susanne S. Scherrer
Abstract Objectives: Compare failure modes and fracture origins using fractography on recovered clinically fractured parts of indirect resin composite endocrowns and overlay restorations on endodontically treated teeth (ETT). Methods: Four endocrowns (3 molars, 1 premolar) and one overlay (molar) adhesively luted on ETT were recovered after fracturing during function. The time in service ranged between 4 and 48 months. The composite materials were (i) CAD/CAM LAVA Ultimate (N = 1), (ii) Premise Indirect (N = 2), and (iii) Colombus (N = 2). Fractography was performed by means of digital microscopy and SEM. Occlusal surfaces were checked for signs of fatigue degradation and contact wear. Cuspal plane angles were measured from profiles obtained from 3D digital microscope images with respect to the horizontal plane of the occlusal central crown groove. Results: All five cases showed a wedge-opening mode I fracture, splitting the crown and tooth in two parts through the crown's central groove. Classic brittle fracture features (arrest lines, twist and wake hackle) were easily identified on the fracture surfaces. Multiple origins were located along the central groove in conjunction with the presence of fatigue cracks. Contact wear surfaces showed pitting and cracking. Cuspal plane angles were around 30–35°, except a 50° palatal cusp slope for the Lava Ultimate overlay. Significance: Fractography on clinical fractures of resin composites was enlightening. Occlusal surface fatigue degradation from cyclic loading, mode I fracture from applied mastication forces on cuspal planes, and stress concentration within the crown's central groove, indicate limitations of use of these materials for endocrowns in posterior teeth.
Date 2021-06
Extra Publisher: Elsevier Inc. PMID: 33627233
Volume 37
Pages e341–e359
Publication Dental Materials
DOI 10.1016/j.dental.2021.02.002
Issue 6
ISSN 01095641

Date Added 1/26/2024, 8:45:05 AM
Modified 1/26/2024, 8:45:05 AM

Tags:

Wear, Clinical, Composite, Crown, Fatigue, Fractography, Fracture, Mode I, Restoration

---

Fracture resistance of monolithic zirconia molar crowns with reduced thickness

**Type**	Journal Article
**Author**	Keisuke Nakamura
**Author**	Akio Harada
**Author**	Ryoichi Inagaki
**Author**	Taro Kanno
**Author**	Yoshimi Niwano
**Author**	Percy Milleding
**Author**	Ulf Örtengren
**Date**	2015-11
**Volume**	73
**Pages**	602–608
**Publication**	Acta Odontologica Scandinavica
**DOI**	10.3109/00016357.2015.1007479
**Issue**	8
**ISSN**	0001-6357
**Date Added**	1/26/2024, 8:45:06 AM
**Modified**	1/26/2024, 8:45:06 AM

Fracture Resistance of Repaired 5Y-PSZ Zirconia Crowns after Endodontic Access

Type Journal Article
Author Andreas Greuling
Author Mira Wiemken
Author Christoph Kahra
Author Hans Jürgen Maier
Author Michael Eisenburger
Abstract <p>This study analyzed the fracture load before and after a chewing simulation of zirconia crowns that were trepanned and repaired using composite resin. Overall, 3 groups with 15 5Y-PSZ crowns in each group were tested. For group A, the fracture load of the unmodified crowns was evaluated. For group B, the crowns were trepanned and repaired using composite resin, also followed by a fracture test. For group C, crowns were prepared like in group B but received thermomechanical cycling before the final fracture tests. Furthermore, scanning electron microscopy (SEM) and X-ray microscopy (XRM) analysis were performed for group C. The mean fracture loads and standard deviation were 2260 N ± 410 N (group A), 1720 N ± 380 N (group B), and 1540 N ± 280 N (group C). Tukey-Kramer multiple comparisons showed a significant difference between groups A and B (p < 0.01) and groups A and C (p < 0.01). After ageing, surface fissures were detected via SEM, but no cracks that reached from the occlusal to the inner side of the crown were detected via XRM. Within the limitations of this study, it can be stated that trepanned and composite-repaired 5Y-PSZ crowns show lower fracture loads than 5Y-PSZ crowns without trepanation.</p>
Date 2023-03
Volume 11
Pages 76

Publication Dentistry Journal
DOI 10.3390/dj11030076
Issue 3
ISSN 2304-6767
Date Added 1/26/2024, 8:45:08 AM
Modified 1/26/2024, 8:45:08 AM

Full-Crown Versus Endocrown Approach: A 3D-Analysis of Both Restorations and the Effect of Ferrule and Restoration Material.

Type Journal Article
Author João Paulo Mendes Tribst
Author Amanda Maria de Oliveira Dal Piva
Author Niek de Jager
Author Marco Antonio Bottino
Author Paul de Kok
Author Cornelis Johannes Kleverlaan
Abstract PURPOSE To assess stress distribution in full-crowns with a composite buildup and endocrowns under axial or oblique loads, both with different ferrules (1 or 2 mm) and ceramic materials (glass ceramic or hybrid ceramic). MATERIALS AND METHODS Sixteen models were analyzed with finite element analysis. No-separation contacts were considered between restoration/resin cement and resin cement/tooth. The contact between the fixation cylinder and the root was considered perfectly bonded. The axial load was applied to the occlusal surface and the oblique load was applied to the buccal cusp. The resulting tensile stresses were shown for the crown, the cement layer and the tooth. RESULTS Almost all factors influenced the stress distribution significantly in the crown and the cement layer, as well as the tooth. The only exception was found under oblique loading by the restoration material and the type of crown that were of no significant influence on the stress distribution in the tooth. CONCLUSIONS Under axial load, the endocrown showed the least tensile stresses in the tooth, but under oblique loads, the full-crown showed less tensile stresses than the endocrown. With the hybrid ceramic material, lower stresses were found in the crown, but higher stresses were present in the cement layer. The 2 mm ferrule is beneficial for reducing the resulting tensile stresses in all modalities.
Date 2021-04
URL http://www.ncbi.nlm.nih.gov/pubmed/32856743
Extra Publisher: Blackwell Publishing Inc. PMID: 32856743
Volume 30
Pages 335–344
Publication Journal of prosthodontics : official journal of the American College of Prosthodontists
DOI 10.1111/jopr.13244
Issue 4
ISSN 1532-849X
Date Added 1/26/2024, 8:45:04 AM
Modified 1/26/2024, 8:45:04 AM

Tags:

CAD/CAM, endocrown, endodontically treated teeth, finite element analysis, mandibular molar, preparation features

---

Influence of a cylindrical crosshead on shear bond testing of composite-tooth interfaces

Type Journal Article
Author Chris S. Ivanoff

Author Timothy L. Hottel
Author Franklin Garcia-Godoy
Date 2018-11
Volume 37
Pages 865–873
Publication Dental Materials Journal
DOI 10.4012/dmj.2017-348
Issue 6
ISSN 0287-4547
Date Added 1/26/2024, 8:45:07 AM
Modified 1/26/2024, 8:45:07 AM

Influence of different surface treatments and universal adhesives on the repair of CAD-CAM composite resins: An in vitro study

Type Journal Article
Author Soner Sismanoglu
Author Zuhal Yildirim-Bilmez
Author Aysegul Erten-Taysi
Author Pınar Ercal
Date 2020-08
Volume 124
Pages 238.e1–238.e9
Publication The Journal of Prosthetic Dentistry
DOI 10.1016/j.prosdent.2020.02.029
Issue 2
ISSN 00223913
Date Added 1/26/2024, 8:45:05 AM
Modified 1/26/2024, 8:45:05 AM

Influence of different universal adhesives on the repair performance of hybrid CAD-CAM materials

Type Journal Article
Author Gülbike Demirel
Author İsmail Hakkı Baltacıoğlu
Date 2019
Volume 44
Publication Restorative Dentistry & Endodontics
DOI 10.5395/rde.2019.44.e23
Issue 3
ISSN 2234-7658
Date Added 1/26/2024, 8:45:08 AM
Modified 1/26/2024, 8:45:08 AM

Influence of finishing time on marginal sealing ability of new generation composite bonding systems

**Type**	Journal Article
**Author**	A. U. J. Yap
**Author**	H. Q. Ang
**Author**	K. C. Chong
**Date**	1998-11
**Volume**	25
**Pages**	871–876
**Publication**	Journal of Oral Rehabilitation
**DOI**	10.1046/j.1365-2842.1998.00316.x
**Issue**	11
**ISSN**	0305-182X
**Date Added**	1/26/2024, 8:45:04 AM
**Modified**	1/26/2024, 8:45:04 AM

Influence of Simulated Oral Conditions on Different Pretreatment Methods for the Repair of Glass-Ceramic Restorations.

**Type**	Journal Article
**Author**	Benedikt Höller
**Author**	Renan Belli
**Author**	Anselm Petschelt
**Author**	Ulrich Lohbauer
**Author**	José Ignacio Zorzin

Abstract PURPOSE The present study investigated the influence of simulated intraoral conditions (increased temperature and humidity) on two different surface pretreatment methods to repair a lithium-disilicate glass-ceramic (LDS). MATERIALS AND METHODS A total of 540 rectangular lithium-disilicate glass-ceramic bars were manufactured (3 x 7 x 9 mm; IPS e.max CAD, Ivoclar Vivadent). Further specimen preparation was performed in an incubator with controlled relative humidity (RH) and temperature to simulate three different environmental settings: laboratory conditions (LC, n = 180, 23°C, 50% RH), rubber-dam conditions (RC, n = 180, 30°C, 50% RH) or oral conditions (OC, n = 180, 32°C, 95 ± 5% RH). One-third of the bars under each condition (n = 60) were grit blasted (GBL) with alumina (35 µm at 1 bar pressure for 10 s and a working distance of 4 ± 1 cm) and primed (60 s, Monobond Plus, Ivoclar Vivadent). Another third (n = 60) were pretreated with a self-etching glass-ceramic primer (MEP, Monobond Etch & Prime, Ivoclar Vivadent). One group without surface pretreatment (n = 60, NoPT) served as a control. All pretreated surfaces were coated with Heliobond (Ivoclar Vivadent). Two bars from the same pretreatment method were luted perpendicular to each other with a resin composite to form a square adhesion area of 9 mm2 (TetricEvo Ceram, Ivoclar Vivadent), and light cured for 20 s on each side (1200 mW/cm2, Bluephase 20i, Ivoclar Vivadent). All specimens were stored for 24 h in distilled water at 37°C. Half of the specimens from each environmental setting and pretreatment method (n = 15) were thermocycled (TC, 5000 cycles, 5/55°C, 30-s dwell time), and tensile bond strength (TBS) testing was performed for all groups using an x-bar rope-assisted set-up. Data were statistically analyzed using two-way ANOVA (a = 0.05) with Bonferroni adjustment. RESULTS Regardless of the environmental and storage conditions (24 h or TC), MEP showed a significantly higher mean TBS than GBL. A decrease in TBS was recorded in specimens under OC compared to RC and LC for both pretreatment methods independent of the storage condition. No significant difference in mean TBS was found between RC and LC within the MEP pretreatment group for the 24 h stored and thermocycled specimens. For all MEPs and GBLs, TC reduced the mean TBS in all environmental conditions. The NoPT groups showed no adhesion regardless of environmental or storage conditions. CONCLUSIONS Increased temperature and high humidity significantly

reduced TBS. However, MEP was less sensitive to environmental influences than GBL, which makes it a promising candidate for intraoral ceramic repair. These findings suggest that clinical intraoral repair of lithium-disilicate glass-ceramics should be performed using a rubber-dam, primarily when using GBL.

Date 2022-03
URL http://www.ncbi.nlm.nih.gov/pubmed/35227047
Extra PMID: 35227047
Volume 24
Pages 57–66
Publication The journal of adhesive dentistry
DOI 10.3290/j.jad.b2701717
Issue 1
ISSN 1757-9988
Date Added 1/26/2024, 8:45:07 AM
Modified 1/26/2024, 8:45:07 AM

Influence of Surface Conditioning Protocols on Reparability of CAD/CAM Zirconia-reinforced Lithium Silicate Ceramic

Type Journal Article
Author Rana Al-Thagafi
Author Walid Al-Zordk
Author Samah Saker
Abstract Purpose: To test the effect of surface conditioning protocols on the reparability of CAD/CAM zirconia-reinforced lithium silicate ceramic compared to lithium-disilicate glass ceramic. Materials and Methods: Zirconia-reinforced lithium silicate ceramic (Vita Suprinity) and lithium disilicate glass-ceramic blocks (IPS e.max CAD) were categorized into four groups based on the surface conditioning protocol used. Group C: no treatment (control); group HF: 5% hydrofluoric acid etching for 60 s, silane (Monobond-S) application for 60 s, air drying; group HF-H: 5% HF acid etching for 60 s, application of silane for 60 s, air drying, application of He-liobond, light curing for 20 s; group CO: sandblasting with CoJet sand followed by silanization. Composite resin (Tet-ric Evo Ceram) was built up into 4 x 6 x 3 mm blocks using teflon molds. All specimens were subjected to thermocycling (5000x, 5°C to 55ºC). The microtensile bond strength test was employed at a crosshead speed of 1 mm/min. SEM was employed for evaluation of all the debonded microbars, the failure type was categorized as either adhesive (failure at adhesive layer), cohesive (failure at ceramic or composite resin), or mixed (failure between adhesive layer and substrate). Two-way ANOVA and the Tukey's HSD post-hoc test were applied to test for significant differences in bond strength values in relation to different materials and surface pretreatment ($p < 0.05$). Results: The highest microtensile repair bond strength for Vita Suprinity was reported in group CO (33.1 ± 2.4 MPa) and the lowest in group HF (27.4 ± 4.4 MPa). Regarding IPS e.max CAD, group CO showed the highest (30.5 ± 4.9 MPa) and HF the lowest microtensile bond strength (22.4 ± 5.7 MPa). Groups HF, HF-H, and CO showed statistically significant differences in terms of all ceramic types used ($p < 0.05$). The control group showed exclusively adhesive failures, while in HF, HF-H, and CO groups, mixed failures were predominant. Conclusions: Repair bond strength to zirconia-reinforced lithium silicate ceramics and lithium-disilicate glass ceramic could be improved when ceramic surfaces are sandblasted with CoJet sand followed by silanization.

Date 2016
Volume 18
Pages 135–141
Publication J Adhes Dent
DOI 10.3290/j.jad.a35909
Issue 2
Date Added 1/26/2024, 8:45:10 AM
Modified 1/26/2024, 8:45:10 AM

Tags:

adhesion, ceramic, intraoral repair

Influence of surface wetness on bonding effectiveness of universal adhesives in etch-and-rinse mode

Type Journal Article
Author Akimasa Tsujimoto
Author Yusuke Shimatani
Author Kie Nojiri
Author Wayne W. Barkmeier
Author Mark D. Markham
Author Toshiki Takamizawa
Author Mark A. Latta
Author Masashi Miyazaki
Date 2018-12
Pages eos.12596
Publication European Journal of Oral Sciences
DOI 10.1111/eos.12596
ISSN 0909-8836
Date Added 1/26/2024, 8:45:04 AM
Modified 1/26/2024, 8:45:04 AM

Intraoral Repair of Direct and Indirect Restorations: Procedures and Guidelines

Type Journal Article
Author BAC Loomans
Author M Özcan
Abstract <p>The service life of defective direct or indirect restorations could be prolonged by repair or relayering actions where durable adhesion of resin-based composite materials is established for longevity of repairs. The advances in adhesive technologies have introduced several surface conditioning concepts to adhere resin composites onto different restorative materials. The purpose of this report is to summarize reasons for failure, survival of repaired reconstructions, elaborate upon types and mechanisms of available surface conditioning methods, and present operative dentists with practical guidelines for intraoral repair procedures.</p>
Date 2016-09
Volume 41
Pages S68–S78
Publication Operative Dentistry
DOI 10.2341/15-269-LIT
Issue S7
ISSN 0361-7734
Date Added 1/26/2024, 8:45:06 AM
Modified 1/26/2024, 8:45:06 AM

Load-bearing capacity under fatigue and FEA analysis of simplified ceramic restorations supported by Peek or zirconia polycrystals as foundation substrate for implant purposes

Type Journal Article
Author Pablo Machado Soares
Author Ana Carolina Cadore-Rodrigues
Author Alexandre Luiz Souto Borges
Author Luiz Felipe Valandro
Author Gabriel Kalil Rocha Pereira
Author Marília Pivetta Rippe
Date 2021-11
Volume 123
Pages 104760
Publication Journal of the Mechanical Behavior of Biomedical Materials
DOI 10.1016/j.jmbbm.2021.104760
ISSN 17516161
Date Added 1/26/2024, 8:45:05 AM
Modified 1/26/2024, 8:45:05 AM

One-step ceramic primer as surface conditioner: Effect on the load-bearing capacity under fatigue of bonded lithium disilicate ceramic simplified restorations

Type Journal Article
Author Kiara Serafini Dapieve
Author Renan Vaz Machry
Author Rafaela Oliveira Pilecco
Author Cornelis Johannes Kleverlaan
Author Gabriel Kalil Rocha Pereira
Author Andressa Borin Venturini
Author Luiz Felipe Valandro
Abstract © 2020 The aim of the study was to evaluate the effect of a ceramic primer and its increased passive application on the fatigue performance of adhesively cemented lithium disilicate simplified restorations. Ceramic discs (Ø = 10 mm; thickness = 1.0 mm) were submitted to an in-lab simulation of CAD/CAM milling and allocated into 8 groups (n = 15), considering 2 factors: "surface treatment"– PRIMER, only coupling agent application (Monobond N); HF5+PRIMER, 5% hydrofluoric acid and coupling agent; E&P 20s + 40s and E&P 20s+5min, ceramic etching/priming (Monobond Etch & Prime, E&P) for 20 s of active application followed by 40 s or 5 min of passive application, respectively; and "aging condition"– baseline, storage for 24 h to 5 days; aged, storage for 90 days +12,000 thermal cycles. Adhesive cementation (Multilink N) was performed onto epoxy discs (Ø = 10 mm; thickness = 2 mm) and the cemented assemblies were subjected to step-stress fatigue tests (initial load of 200 N; step-size of 50 N; 10,000 cycles per step; 20 Hz). The results showed that the groups had similar fatigue performance in the baseline condition (except for E&P 20s+5min: 940.0 N; 123,000 cycles > PRIMER: 786.7 N; 92,333 cycles). When aged, the PRIMER group presented the worst fatigue performance (480.8 N; 31,154 cycles) compared to the other groups (810.0–840.0 N; 97,000–103,000 cycles). In addition, only the PRIMER treatment showed unstable fatigue performance (baseline > aged). Therefore, ceramic surface treatment promoting micromechanical interlocking and chemical bonds is mandatory for stable fatigue performance of adhesively cemented lithium disilicate restorations. The one-step ceramic primer/conditioner promoted similar fatigue performance to the 5% hydrofluoric acid + coupling agent, but increased E&P etching time did not improve the fatigue behavior.
Date 2020
Extra Publisher: Elsevier Ltd

**Volume**	104
**Pages**	103686
**Publication**	Journal of the Mechanical Behavior of Biomedical Materials
**DOI**	10.1016/j.jmbbm.2020.103686
**Date Added**	1/26/2024, 8:45:08 AM
**Modified**	1/26/2024, 8:45:08 AM

Tags:

Computer aided design/computer aided machining, Etching, Mechanical phenomena, Survival probability, Topographical changes, Weibull analysis

---

Possible hazardous effects of hydrofluoric acid and recommendations for treatment approach: a review

**Type**	Journal Article
**Author**	Mutlu Özcan
**Author**	Arezo Allahbeickaraghi
**Author**	Mine Dündar
**Date**	2012-02
**Volume**	16
**Pages**	15–23
**Publication**	Clinical Oral Investigations
**DOI**	10.1007/s00784-011-0636-6
**Issue**	1
**ISSN**	1432-6981
**Date Added**	1/26/2024, 8:45:06 AM
**Modified**	1/26/2024, 8:45:06 AM

Repair bond strength and nanoleakage of artificially aged CAD-CAM composite resin

**Type**	Journal Article
**Author**	Carmen Arpa
**Author**	Laura Ceballos
**Author**	María Victoria Fuentes
**Author**	Jorge Perdigão
**Date**	2019-03
**Volume**	121
**Pages**	523–530
**Publication**	The Journal of Prosthetic Dentistry
**DOI**	10.1016/j.prosdent.2018.05.013
**Issue**	3
**ISSN**	00223913
**Date Added**	1/26/2024, 8:45:09 AM
**Modified**	1/26/2024, 8:45:09 AM

Repair bond strength and surface topography of resin-ceramic and ceramic restorative blocks treated by laser and conventional surface treatments

Type Journal Article
Author Yusuf Bayraktar
Author Merve Arslan
Author Zulfikar Demirtag
Abstract This study intended to compare the repair bond strength of computer-aided design/computer-aided manufacturing (CAD/CAM) blocks consisting of resin and feldspathic ceramics following different surface treatments using the microtensile bond strength (μTBS) test. Ten specimens were prepared with 4 mm height for Vita Enamic (VE), Lava Ultimate (LU), Vita Mark II (VM), and thermocycled (10,000 cycle, 5–55°C). Each material was categorized into one of five subgroups according to following surface treatments: (a) bur grinding (BG), (b) hydrofluoric acid etching (HF), (c) neodymium-doped yttrium aluminum garnet (Nd:YAG or NY), (d) erbium-doped yttrium aluminum garnet (Er:YAG or EY), and (e) erbium, chromium-doped yttrium, scandium, gallium, and garnet (Er,Cr:YSGG or ECY) laser conditioning. After surface treatment procedures, specimens were properly restored to 4 mm high with a micro-hybrid composite resin. Bar specimens (1 × 1 × 8 mm) were obtained using a low-speed cutting machine and then thermocycled (10,000 cycle, 5–55°C). The μTBS was tested at 1 mm/min crosshead speed, and failure modes were evaluated. Data were analyzed with two-way ANOVA and post hoc Tukey tests. LU-BG showed significantly higher μTBS (32.94 ± 5.80 MPa) compared to LU-laser groups ($p < .05$). VE-BG showed significantly higher μTBS (22.06 ± 4.26 MPa) compared to other VE groups ($p < .05$). Among the laser groups, the NY laser produced the lowest ($p < .05$) μTBS for LU (13.42 ± 3.44 MPa) and VE (2.27 ± 0.85 MPa), while EY showed the highest ($p < .05$). Laser-treated VM groups were all prefailured. VM-HF produced a higher μTBS (18.73 ± 3.75 MPa) than VM-BG (5.05 ± 1.76 MPa) ($p < .05$).
Date 2021-06
Extra Publisher: John Wiley and Sons Inc PMID: 33615613
Volume 84
Pages 1145–1154
Publication Microscopy Research and Technique
DOI 10.1002/jemt.23672
Issue 6
ISSN 10970029
Date Added 1/26/2024, 8:45:08 AM
Modified 1/26/2024, 8:45:08 AM

Tags:

Er:YAG laser, microtensile bond strength, Cr:YSGG laser, Er, Nd:YAG laser, scanning electron microscopy

---

Repair Bond Strength of a CAD/CAM Nanoceramic Resin and Direct Composite Resin: Effect of Aging and Surface Conditioning Methods.

Type Journal Article
Author Dayanne Monielle Duarte Moura
Author Amanda Maria de Oliveira Dal Piva
Author Ana Beatriz do Nascimento Januário
Author Aretha Heitor Verissímo
Author Marco Antonio Bottino
Author Mutlu Özcan
Author Rodrigo Othávio Assunção Souza

Abstract PURPOSE To evaluate the effect of surface conditioning methods and aging on the repair bond strength between resin composite and nanoceramic CAD/CAM resin. MATERIALS AND METHODS Twenty-four blocks of nanoceramic CAD/CAM resin (NCR) (Lava Ultimate, 3M Oral Care) (10 x 5 x 2 mm3) and resin composite (Filtek Z350, 3M Oral Care) (RC) were made, embedded in acrylic resin, polished (#600, #800, #1200) and randomly divided into 8 groups (n = 12 each) according to surface conditioning methods (air abrasion with 30-µm CoJet [CJ] or air abrasion with 50-µm Al2O3 [AB]) and aging prior to repair (without aging, 24 h in water at 37°C; with aging 6 months in water at 37°C). The blocks were air abraded (20 s, 2.5 bar, 10 mm) using a standardized device. A layer of adhesive resin (Scotchbond Universal) was applied (20 s) and photopolymerized for 20 s. RC cylinders (Ø = 2 mm; h = 2 mm) were then bonded to the NCR substrates using a Teflon matrix and photopolymerized for 40 s. All specimens were thermocycled (10,000 cycles, 5°C-55°C) and submitted to the shear bond test (50 kgf, 0.5 mm/min) to measure repair strength. Data (MPa) were analyzed using 3-way ANOVA and Tukey's test ($\alpha = 0.05$). Failure analysis was performed using stereomicroscopy (20X). RESULTS ANOVA revealed a significant effect of only the "material" factor ($p = 0.00$). The group NCR6mCJ presented bond strengths (29.37 ± 5.41) which were significantly higher than those of the NCR24hCJ (20.88 ± 5.74) and RC groups ($p < 0.05$). The group RC24hCJ (19.71 ± 4.21) presented the lowest shear bond strength ($p < 0.05$). Failure analysis revealed predominantly type B mixed failures (adhesive+cohesive in the substrate material) except for the groups NCR24hCJ and NCR6mAB, where mainly type C failure (adhesive+cohesive at the RC) was observed. CONCLUSION Air abrasion with Al2O3 particles or silicatization with CoJet followed by adhesive resin application are effective surface conditioning methods for the repair of nanoceramic CAD/CAM resin with resin composite.

Date 2020

Extra PMID: 32435768

Volume 22

Pages 275–283

Publication The journal of adhesive dentistry

DOI 10.3290/j.jad.a44551

Issue 3

ISSN 1757-9988

Date Added 1/26/2024, 8:45:04 AM

Modified 1/26/2024, 8:45:04 AM

Repair bond strength of dental computer-aided design/computer-aided manufactured ceramics after different surface treatments

Type Journal Article

Author Lulwa AL-Turki

Author Yasser Merdad

Author Tariq A. Abuhaimed

Author Dania Sabbahi

Author Mazen Almarshadi

Author Raghad Aldabbagh

Abstract Objective: To evaluate the microtensile bond strength of four dental computer-aided design/computer-aided manufactured (CAD/CAM) ceramics after application of four different surface treatments. Materials and methods: Four dental CAD/CAM ceramics were tested: feldspathic ceramic (VITABLOCKS-Mark II), polymer-infiltrated ceramic network (VITA ENAMIC), zirconia-reinforced lithium silicate (VITA SUPRINITY), and yttria-stabilized zirconia (VITA YZ T). Four surface treatments were applied: no treatment, 5% hydrofluoric acid-etching, airborne particle abrasion, and tribochemical silica coating. The ceramic blocks were repaired with nanohybrid composite (Tetric N-Collection). Sixteen test groups of 12 specimens were prepared. After thermocycling, microtensile bond testing was performed. The microtensile strengths values were statistically analyzed using two-way analysis of variance and Tukey's post-hoc test. Results: Repaired feldspathic and resin polymer-infiltrated ceramic network ceramics demonstrated superior microtensile bond strengths compared to zirconia-

reinforced lithium silicate and yttria-stabilized zirconia. Etched feldspathic and polymer-infiltrated ceramic network ceramics had higher bond strength than the untreated groups. Surface treatments did not affect the bond strength of zirconia-reinforced lithium silicate and yttria-stabilized zirconia with the exception of etching, which reduced the bond strength of yttria-stabilized zirconia. Conclusion: Feldspathic ceramic and polymer-infiltrated ceramic network were repaired with dental composite after surface etching with hydrofluoric acid. Repair of zirconia-reinforced lithium silicate and yttria-stabilized zirconia did not demonstrate promising results. Clinical significance: Repair of feldspathic ceramic and polymer-infiltrated ceramic network restorations may be a cost-effective means to promote the longevity of dental restorations. However, zirconia and zirconia-reinforced lithium disilicate restorations do not offer such an option.

Date 2020-10

Extra Publisher: Blackwell Publishing Ltd PMID: 32886852

Volume 32

Pages 726–733

Publication Journal of Esthetic and Restorative Dentistry

DOI 10.1111/jerd.12635

Issue 7

ISSN 17088240

Date Added 1/26/2024, 8:45:09 AM

Modified 1/26/2024, 8:45:09 AM

Tags:

hydrofluoric acid, microtensile bond strength, CAD/CAM ceramics, airborne particle abrasion, repair, tribochemical silicoating

---

Repair bond strength of different CAD-CAM ceramics after various surface treatments combined with laser irradiation

Type Journal Article

Author Seda Üstün Aladağ

Author Elif Aydoğan Ayaz

Abstract The aim of this study was to evaluate the effect of different surface treatments combined with laser irradiation on the shear bond strength of different CAD-CAM ceramics to composite resin. A total of hundred forty-seven ceramic specimens with thicknesses of 2.5 mm were prepared from three different CAD-CAM ceramics (an yttrium oxide partially stabilized tetragonal zirconia polycrystal (Y-TZP); a zirconia-reinforced lithium silicate glass ceramic (ZLS); and a lithium disilicate–strengthened lithium aluminosilicate glass ceramic (LD-LAS)) and subjected to seven groups of treatment ($n = 7$): (1) control (no treatment), (2) Er:YAG laser irradiation, (3) Nd:YAG laser irradiation, (4) etching with hydrofluoric acid (HFA), (5) Er:YAG + HFA, (6) Nd:YAG + HFA, and (7) sandblasting. After surface treatment procedures, a ceramic primer (Clearfil Ceramic Primer Plus, Kuraray, Japan) was applied to the ceramics. Bonding agent (Single Bond Universal Adhesive, 3 M ESPE, USA) was then applied, and the composite resin (Estelite Sigma Quick, Kuraray, Japan) was layered on the ceramic surfaces. The shear bond strength test was performed using a universal testing machine at a load of 0.5 mm/min. Data were analyzed by 2-way analysis of variance (ANOVA), and the Bonferroni correction was used for pairwise comparisons ($\alpha = 0.05$). Compared to the bond strength of the control group, irradiation by Er:YAG and Nd:YAG lasers alone improved the bond strength of the composite resin to the Y-TZP ($P < 0.001$) but did not change the bond strength of composite resin to the ZLS and LD-LAS ($P > 0.05$). Compared to the bond strength of the control group, etching with HFA alone increased the bond strength of the composite resin to the ZLS and LD-LAS ($P < 0.001$) but did not affect the bond strength of the composite resin to the Y-TZP ($P > 0.05$). The highest bond strength of ZLS was obtained using HFA + Er:YAG, and the highest bond strength for LD-LAS was obtained using HFA + Nd:YAG. It was concluded that Er:YAG and Nd:YAG laser treatments presented the highest repair bond strength between the composite resin and Y-

TZP ceramics. Er:YAG and Nd:YAG laser treatments in conjuction with HFA presented the highest repair bond strength between the composite resin and the glassy ceramics, ZLS, and LD + LAS.

**Date**	2023-12
**Extra**	Publisher: Springer Science and Business Media Deutschland GmbH PMID: 36689017
**Volume**	38
**Publication**	Lasers in Medical Science
**DOI**	10.1007/s10103-023-03715-3
**Issue**	1
**ISSN**	1435604X
**Date Added**	1/26/2024, 8:45:10 AM
**Modified**	1/26/2024, 8:45:10 AM

Tags:

Shear strength, Ceramics, Er:YAG laser, Nd:YAG laser, CAD-CAM, Dental acid etching

---

Repair bond strength of resin composite to restorative materials after short- and long-term storage

**Type**	Journal Article
**Author**	Simon Flury
**Author**	Fabrice A. Dulla
**Author**	Anne Peutzfeldt
**Date**	2019-09
**Volume**	35
**Pages**	1205–1213
**Publication**	Dental Materials
**DOI**	10.1016/j.dental.2019.05.008
**Issue**	9
**ISSN**	01095641
**Date Added**	1/26/2024, 8:45:08 AM
**Modified**	1/26/2024, 8:45:08 AM

Repair bond strength of resin composite to three aged CAD/CAM blocks using different repair systems

**Type**	Journal Article
**Author**	Pinar Gul
**Author**	Latife Altınok-Uygun
**Date**	2020
**Volume**	12
**Pages**	131
**Publication**	The Journal of Advanced Prosthodontics
**DOI**	10.4047/jap.2020.12.3.131
**Issue**	3
**ISSN**	2005-7806
**Date Added**	1/26/2024, 8:45:07 AM
**Modified**	1/26/2024, 8:45:07 AM

Repair bond strengths of non-aged and aged resin nanoceramics

**Type**	Journal Article
**Author**	Meryem Gülce Subaşı
**Author**	Gülce Alp
**Date**	2017
**Volume**	9
**Pages**	364
**Publication**	The Journal of Advanced Prosthodontics
**DOI**	10.4047/jap.2017.9.5.364
**Issue**	5
**ISSN**	2005-7806
**Date Added**	1/26/2024, 8:45:05 AM
**Modified**	1/26/2024, 8:45:05 AM

Repair may increase survival of direct posterior restorations – A practice based study

**Type**	Journal Article
**Author**	Luciano Casagrande
**Author**	Mark Laske
**Author**	Ewald M. Bronkhorst
**Author**	Marie Charlotte D.N.J.M. Huysmans
**Author**	Niek J.M. Opdam
**Date**	2017-09
**Volume**	64
**Pages**	30–36
**Publication**	Journal of Dentistry
**DOI**	10.1016/j.jdent.2017.06.002
**ISSN**	03005712
**Date Added**	1/26/2024, 8:45:08 AM
**Modified**	1/26/2024, 8:45:08 AM

Repair of Aged Polymer-Based CAD/CAM Ceramics Treated with Different Bonding Protocols

**Type**	Journal Article
**Author**	Elif Bayazıt
**Date**	2021-05
**Volume**	34
**Pages**	357–364
**Publication**	The International Journal of Prosthodontics
**DOI**	10.11607/ijp.6660
**Issue**	3
**ISSN**	08932174
**Date Added**	1/26/2024, 8:45:09 AM
**Modified**	1/26/2024, 8:45:09 AM

Repair strength and surface topography of lithium disilicate and hybrid resin ceramics with LLLT and photodynamic therapy in comparison to hydrofluoric acid

**Type**	Journal Article
**Author**	Ahmed M. Maawadh
**Author**	Thamer Almohareb
**Author**	Rana S. Al-Hamdan
**Author**	Modhi Al Deeb
**Author**	Mustafa Naseem
**Author**	Aasem M. Alhenaki
**Author**	Fahim Vohra
**Author**	Tariq Abduljabbar
**Abstract**	Aim: The aim was to compare the repair bond strength and surface topography of lithium disilicate ceramics (LDC) and hybrid resin ceramics (HRC) using different surface conditioning treatments [low level laser therapy (LLLT), photodynamic therapy (PDT), hydrofluoric acid (HF) with silane and air abrasion (AA) and silane]. Material and method: Sixty specimens each of LDC and HRC were used. Discs were prepared for each group (6 × 2 mm), conditioned using different regimes. Specimens in group 1 and 5 were laser irradiated using Er,Cr:YSGG (ECYL), group 2 and 6 were conditioned using methylene blue photosensitizer (PDT), group 3 and 7 surface was treated with hydrofluoric acid and silane (HFA-S), group 4 and 8 conditioned with Al2O3 air abrasion and silane (AA-S). A Porcelain Repair Kit was used according to manufacturer recommendation in all samples. Peak universal bond adhesive was rubbed on ceramic surface and then bonded with composite resin. For shear bond strength testing the specimens were placed in a universal testing machine. A stereomicroscope at 40x magnification was used to analyse failure pattern. Five specimens in each group after surface treatment were evaluated for surface changes and topography using scanning electron microscopy. The mean repair bond strength was calculated using ANOVA and Tukey's post hoc test at a significance level of ($p < 0.05$). Result: The highest repair bond strength was observed in group 3 (LDC) (20.57 ± 3.58 MPa) (HFA-S), whereas, the lowest score was displayed in Group 2 (LDC) using methylene blue photosensitizer (MBPS) (12.18 ± 1.08 MPa). Similarly, in HRC the highest repair SBS was presented in group 8 (AA-S) (20.52 ± 2.51 MPa) and the lowest SBS values were exhibited by PDT treated group 6 (13.22 ± 0.62 MPa). Conclusion: A combination of mechanical and chemical surface treatments should be used in order to achieve adequate repair bond strength between resin composites and ceramic interface of LDC and HRC.
**Date**	2020
**Extra**	Publisher: SAGE Publications Ltd PMID: 33270475
**Volume**	18
**Publication**	Journal of Applied Biomaterials and Functional Materials
**DOI**	10.1177/2280800020966938
**ISSN**	22808000
**Date Added**	1/26/2024, 8:45:06 AM
**Modified**	1/26/2024, 8:45:06 AM

Tags:

conditioning treatments, hybrid resin ceramic, lithium disilicate, Repair bond strength

---

Repair Success of Two Innovative Hybrid Materials as a Function of Different Surface Treatments

**Type**	Journal Article
**Author**	Numan Tatar
**Author**	Cagri Ural

Date 2018-05
Volume 31
Pages 267–270
Publication The International Journal of Prosthodontics
DOI 10.11607/ijp.5581
Issue 3
ISSN 08932174
Date Added 1/26/2024, 8:45:05 AM
Modified 1/26/2024, 8:45:05 AM

Retrospective survival analysis of 261 lithium disilicate crowns in a private general practice.

Type Journal Article
Author Marco Valenti
Author Alessandro Valenti
Abstract OBJECTIVES This retrospective study evaluated the clinical performance and long-term survival of glass-ceramic lithium disilicate all-ceramic restorations in anterior and posterior areas over a 10-year period. METHOD AND MATERIALS Following a consistent protocol over 10 years, 261 lithium disilicate crowns (IPS Empress 2, Ivoclar Vivadent), including 101 anterior (38.7%) and 160 posterior (61.3%), were cemented using an adhesive technique and observed by the clinician who prepared and luted them. The data were analyzed using the Kaplan-Meier test. The clinical evaluation adopted the California Dental Association (CDA) modified criteria after recalling all patients between January and May 2008. RESULTS Six crowns failed and were replaced, 4 due to chipping and 2 due to core fracture. The overall survival rate was 95.5%. CONCLUSION In this retrospective evaluation, lithium disilicate ceramic restorations had a low clinical failure rate after up to 120 months.
Date 2009
Extra PMID: 19626232
Volume 40
Pages 573–9
Publication Quintessence international (Berlin, Germany : 1985)
Issue 7
ISSN 1936-7163
Date Added 1/26/2024, 8:45:04 AM
Modified 1/26/2024, 8:45:04 AM

Same, same, but different? A systematic review of protocols for restoration repair

Type Journal Article
Author Philipp Kanzow
Author Annette Wiegand
Author Falk Schwendicke
Author Gerd Göstemeyer
Date 2019-07
Volume 86
Pages 1–16
Publication Journal of Dentistry
DOI 10.1016/j.jdent.2019.05.021
ISSN 03005712
Date Added 1/26/2024, 8:45:07 AM

Modified 1/26/2024, 8:45:07 AM

Self-etching Primers vs Acid Conditioning: Impact on Bond Strength Between Ceramics and Resin Cement

Type Journal Article
Author JPM Tribst
Author LC Anami
Author M Özcan
Author MA Bottino
Author RM Melo
Author GSFA Saavedra
Abstract <p>This study tested whether a self-etching surface agent and the conventional hydrofluoric acid (HF) would provide the same bonding capacity between resin cement and feldspathic (Fd) and lithium disilicate (Ld) ceramics. Ceramic blocks were cut with a low-speed diamond saw with water cooling (Isomet 1000, Buehler, Lake Bluff, IL, USA) into 20 blocks of 5 × 7 × 4 mm, which were ground flat in a polishing machine (EcoMet/AutoMet 250, Buehler) under water cooling. The blocks were randomly divided into eight groups (n=5), according to ceramic type (Ld or Fd), surface conditioning (HF + Monobond Plus or Etch and Prime), and aging by thermocycling (TC or absence-baseline). After 24 hours in 37°C distilled water, blocks were embedded into acrylic resin and 1-mm2 cross-section beams composed of ceramic/cement/composite were obtained. The microtensile test was performed in a universal testing machine (DL-1000, EMIC, São José dos Campos, Brazil; 0.5 mm.min−1, 50 kgf load cell). Bond strength (MPa) was calculated by dividing the load at failure (in N) by the bonded area (mm2). The fractured specimens were examined under stereomicroscopy, and one representative sample of each group was randomly selected before the cementation and was further used for analysis using scanning electron microscopy (SEM) and energy-dispersive x-ray spectroscopy (EDS). The self-etching agent showed the highest bond strength for Fd (24.66±4.5) and Ld (24.73±6.9) ceramics and a decrease in surface wettability. SEM and EDS showed the presence of similar components in the tested materials with different topographies for both. Therefore, the self-etching primer was able to deliver even higher bonding than HF+silane to a resin cement.</p>
Date 2018-07
URL https://meridian.allenpress.com/operative-dentistry/article/43/4/372/194856/Selfetching-Primers-vs-Acid-Conditioning-Impact-on
Volume 43
Pages 372–379
Publication Operative Dentistry
DOI 10.2341/16-348-L
Issue 4
ISSN 0361-7734
Date Added 1/26/2024, 8:45:04 AM
Modified 1/26/2024, 8:45:04 AM

Tags:

Ceramics, Acid Etching, Adhesiveness, Aluminum Silicates, chemistry, Dental, Dental Bonding, Dental Porcelain, Dental Stress Analysis, Electron, Materials Testing, methods, Microscopy, Potassium Compounds, Resin Cements, Scanning, Spectrometry, Surface Properties, Wettability, X-Ray Emission

Shear bond strength of a new self-adhering flowable composite resin for lithium disilicatereinforced CAD/CAM ceramic material

**Type**	Journal Article
**Author**	Ugur Erdemir
**Author**	Hande Sar Sancakli
**Author**	Erkan Sancakli
**Author**	Meltem Mert Eren
**Author**	Sevda Ozel
**Author**	Taner Yucel
**Author**	Esra Yildiz
**Abstract**	Purpose. The purpose of this study was to evaluate and compare the effects of different surface pretreatment techniques on the surface roughness and shear bond strength of a new self-adhering flowable composite resin for use with lithium disilicate-reinforced CAD/CAM ceramic material. Materials and methods. A total of one hundred thirty lithium disilicate CAD/CAM ceramic plates with dimensions of 6 mm × 4 mm and 3 mm thick were prepared. Specimens were then assigned into five groups (n=26) as follows: untreated control, coating with 30 µm silica oxide particles (CojetTM Sand), 9.6% hydrofluoric acid etching, Er:YAG laser irradiation, and grinding with a high-speed fine diamond bur. A self-adhering flowable composite resin (Vertise Flow) was applied onto the pre-treated ceramic plates using the Ultradent shear bond Teflon mold system. Surface roughness was measured by atomic force microscopy. Shear bond strength test were performed using a universal testing machine at a crosshead speed of 1 mm/min. Surface roughness data were analyzed by one-way ANOVA and the Tukey HSD tests. Shear bond strength test values were analyzed by Kruskal-Wallis and Mann-Whitney U tests at α=.05. Results. Hydrofluoric acid etching and grinding with high-speed fine diamond bur produced significantly higher surface roughness than the other pretreatment groups (P < .05). Hydrofluoric acid etching and silica coating yielded the highest shear bond strength values (P < .001). Conclusion. Self-adhering flowable composite resin used as repair composite resin exhibited very low bond strength irrespective of the surface pretreatments used.
**Date**	2014
**Extra**	Publisher: Korean Academy of Prosthodontic
**Volume**	6
**Pages**	434–443
**Publication**	Journal of Advanced Prosthodontics
**DOI**	10.4047/jap.2014.6.6.434
**Issue**	6
**ISSN**	20057814
**Date Added**	1/26/2024, 8:45:08 AM
**Modified**	1/26/2024, 8:45:08 AM

Tags:

CAD/CAM ceramic, Er:YAG laser, Shear bond strength, Surface pretreatments, Tribochemical silica coating, Vertise flow

Shear bond strength of a novel porcelain repair system for different computer-aided design/computer-assisted manufacturing ceramic materials

**Type**	Journal Article
**Author**	M. Karci
**Author**	N. Demir
**Author**	M. Subasi

Author M. Gokkaya

Abstract Objectives: The purpose of this study was to compare the shear bond strength of a novel repair system, Nova Compo SF with Ceramic Repair, Ivoclar, to computer-aided design/computer-assisted manufacturing (CAD/CAM) restorative materials (IPS e.max CAD and Empress CAD). Materials and Methods: The specimens of each CAD/CAM restorative material were randomly divided into two subgroups of nine specimens, using one of two repair systems. All specimens were etched with hydrofluoric acid and rinsed under a water spray for 10 s, then air-dried for 10 s. Next, repair systems were applied according to the manufacturer's instructions. All specimens were stored in distilled water at 37°C for 24 h and then additionally aged for 5000 thermal cycles. A shear bond strength test was performed using a universal testing machine. Each fracture type was examined under a stereomicroscope at ×12.5 magnification. A two-way ANOVA test was used to detect significant differences between the CAD/CAM restorative materials and the composite repair systems. Subgroup analyses were performed using Tukey's honest significant difference. Results: No statistically significant differences were observed between the repair systems ($P = 0.9$). The bond strength values from Empress CAD were statistically higher than those from e.max CAD ($P < 0.05$). Conclusions: Within limitations, SuperFlow may be an alternative to the ceramic repair materials we routinely used in the clinic. Empress CAD can be preferable to e.max CAD in terms of esthetically suitable clinical indications.

Date 2018-04

Extra Publisher: Medknow Publications PMID: 29607866

Volume 21

Pages 507–513

Publication Nigerian Journal of Clinical Practice

DOI 10.4103/njcp.njcp_127_17

Issue 4

ISSN 11193077

Date Added 1/26/2024, 8:45:07 AM

Modified 1/26/2024, 8:45:07 AM

Tags:

Bond strength, computer-aided design/computer-assisted manufacturing, porcelain, repair system

---

Shear Bond Strength of Repair Systems to New CAD/CAM Restorative Materials

Type Journal Article

Author Özlem Üstün

Author Işıl Keçik Büyükhatipoğlu

Author Aslı Seçilmiş

Abstract Purpose: To evaluate the bond strength of repair systems (Ceramic Repair, Clearfil Repair) to computer-aided design/computer-assisted machining (CAD/CAM) restorative materials (IPS e.max CAD, Vita Suprinity, Vita Enamic, Lava Ultimate). Materials and Methods: Thermally aged CAD/CAM restorative material specimens (5000 cycles between 5°C and 55°C) were randomly divided into two groups according to the repair system: Ceramic Repair (37% phosphoric acid + Monobond-S + Heliobond + Tetric N Ceram) or Clearfil Repair (40% phosphoric acid + mixture of Clearfil Porcelain Bond Activator and Clearfil SE Bond Primer + Clearfil SE Bond + Filtek Z250). The resin composite was light-cured on conditioned specimens. All specimens were stored in distilled water at 37°C for 24 hours and then additionally aged for 5000 thermal cycles. The shear bond strength test was performed using a universal testing machine (0.5 mm/min). Two-way ANOVA was used to detect significance differences according to the CAD/CAM material and composite repair system factors. Subgroup analyses were conducted using the least significant difference post-hoc test. Results: The results of two-way ANOVA indicated that bond strength values varied according to the restorative materials ($p < 0.05$). No significant differences were observed between the CAD/CAM restorative materials ($p > 0.05$), except in the Vita Suprinity group ($p < 0.05$). Moreover, no differences were observed

between the repair systems. Conclusions: Both the Clearfil and Ceramic repair systems used in the study allow for successful repairs.

Date 2018-10
Extra Publisher: Blackwell Publishing Inc. PMID: 27880011
Volume 27
Pages 748–754
Publication Journal of Prosthodontics
DOI 10.1111/jopr.12564
Issue 8
ISSN 1532849X
Date Added 1/26/2024, 8:45:04 AM
Modified 1/26/2024, 8:45:04 AM

Tags:

CAD/CAM materials, repair bond strength

Shear bond strengths of aged and non-aged CAD/CAM materials after different surface treatments

Type Journal Article
Author Hamiyet Kilinc
Author Fatma Ayse Sanal
Author Sedanur Turgut
Abstract To assess shear bond strengths (SBS) of resin composites on aged and non-aged prosthetic materials with various surface treatments. MATERIALS AND METHODS. Cerasmart (CE), Vita Enamic (VE), Vita Mark II (VM), and IPS e.max CAD (EC) blocks were sliced, and rectangular-shaped specimens (14 × 12 × 1.5 mm; N = 352) were obtained. Half of the specimens were aged (5000 thermal cycles) for each material. Non-aged and aged specimens were divided into 4 groups according to the surface treatments (control, air abrasion, etching, and laser irradiation; n = 11) and processed for scanning electron microscopy (SEM). The repair procedure was performed after the surface treatments. SBS values and failure types were determined. Obtained data were statistically analyzed ($P \leq .05$). RESULTS. The material type, surface treatment type, and their interactions were found significant with regard to SBS ($P < .001$). Aging also had a significant effect on prosthetic material-resin composite bonding ($P < .001$). SBS values of non-aged specimens ranged from 12.16 to 17.91 MPa, while SBS values of aged specimens ranged from 9.46 to 15.61 MPa. Non-aged VM in combination with acid etching presented the highest score while the control group of aged CE showed the lowest. CONCLUSION. Etching was more effective in achieving durable SBS for VM and EC. Laser irradiation could be considered as an alternative surface treatment method to air abrasion for all tested materials. Aging had significant effect on SBS values generated between tested materials and resin composite. [J Adv Prosthodont 2020;12:273-82]
Date 2020-10
Extra Publisher: Korean Academy of Prosthodontic
Volume 12
Pages 273–282
Publication Journal of Advanced Prosthodontics
DOI 10.4047/jap.2020.12.5.273
Issue 5
ISSN 20057814
Date Added 1/26/2024, 8:45:07 AM
Modified 1/26/2024, 8:45:07 AM

Tags:

Bond strength, Surface treatment, Repair, Computer-aided design and computer-aided manufacturing (CAD/CAM), Laser, Thermal aging

---

Silane adhesion mechanism in dental applications and surface treatments: A review

**Type**	Journal Article
**Author**	Jukka Pekka Matinlinna
**Author**	Christie Ying Kei Lung
**Author**	James Kit Hon Tsoi
**Abstract**	Objective To give a current review of silane adhesion chemistry, applications of silane coupling agents and related surface pretreatment methods in contemporary dentistry. Methods Silane coupling agents are adhesion promoters to chemically unify dissimilar materials used in dentistry. Silanes are very effective in adhesion promotion between resin composites and silica-based or silica-coated indirect restorative materials. It is generally accepted that for non-silica-based restorations, surface pretreatment is a mandatory preliminary step to increase the silica content and then, with help of silane, improve resin bonding. This review discusses the silane-based adhesion chemistry, silane applications in dentistry, surface pretreatment methods, and presents the recent development of silane coupling agents. Results A silane coupling agent is considered a reliable, good adhesion promoter to silica-based (or silica-coated) indirect restorations. Surface pre-treatment steps, e.g., acid etching for porcelain and tribo-chemical silica-coating for metal alloys, is used before silanization to attain strong, durable bonding of the substrate to resin composite. In clinical practice, however, the main problem of resin bonding using silanes and other coupling agents is the weakening of the bond (degradation) in the wet oral environment over time. Significance A silane coupling agent is a justified and popular adhesion promoter (adhesive primer) used in dentistry. The commercial available silane coupling agents can fulfil the requirements in clinical practice for durable bonding. Development of new silane coupling agents, their optimization, and surface treatment methods are in progress to address the long term resin bond durability and are highly important.
**Date**	2018
**URL**	https://doi.org/10.1016/j.dental.2017.09.002
**Extra**	Publisher: The Academy of Dental Materials
**Volume**	34
**Pages**	13–28
**Publication**	Dental Materials
**DOI**	10.1016/j.dental.2017.09.002
**Issue**	1
**Date Added**	1/26/2024, 8:45:06 AM
**Modified**	1/26/2024, 8:45:06 AM

Tags:

Silane, Adhesion chemistry, Bonding, Dental restorative materials, Resin composites

---

Sliding tribological behaviors of Cu and Fe against Si3N 4 under electrolysis

**Type**	Journal Article
**Author**	W Ma
**Author**	B Zhang
**Author**	A Nakajima
**Author**	T Mawatari

Abstract Electrolytic in-Process Dressing (ELID) has been found to have wide application especially in superfine grinding, but its mechanism is still unclarified yet. Simulation experiments were conducted by using an electrolysis friction test machine, which is a ball-on-disk friction machine equipped with an electrolyzing system. Copper, which is one of the most widely used metal bond materials of super abrasive wheels, and iron, one of the most widely used bond materials in ELID, were tested in three different types of electrolyte: tap water, electrolyzed water, and solution of CG-7 to tap water. Experimental results showed that there was almost no detectable wear for both copper and iron when electrolysis was not implemented, and also the material removal was little when only electrolysis was applied. Strong synergistic effect between electrolysis and friction was observed for copper in both tap water and electrolyzed water, and for iron in electrolyzed water and CG-7 solution. The friction coefficient of Si3N4 ball against iron disk was lower than that against copper disk. Copyright © 2013 Japanese Society of Tribologists.

Date 2013
URL https://www.scopus.com/inward/record.uri?eid=2-s2.0-84877856476&doi=10.2474%2Ftrol.8.186&partnerID=40&md5=afdac63d50d772d371e692c626f177ca
Volume 8
Pages 186–191
Publication Tribology Online
DOI 10.2474/trol.8.186
Issue 2
Date Added 1/26/2024, 8:45:04 AM
Modified 1/26/2024, 8:45:04 AM

Tags:

Ball-on-disk friction, Ceramic ball, Ceramic balls, Copper, Electrolysis, Electrolytic in process dressing (ELID), ELID, Friction, Friction coefficients, Grinding (machining), Iron, Silicon, Sliding friction, Synergistic effect, Tribological behaviors, Tribology, Wear, Wear of materials

---

Surface etching and silane heating using Er:YAG and Nd:YAG lasers in dental ceramic luted to human dentin

Type Journal Article
Author Fernanda A. Feitosa
Author João P. M. Tribst
Author Rodrigo M. Araújo
Author César R. Pucci
Date 2021-09
Volume 18
Pages 1408–1416
Publication International Journal of Applied Ceramic Technology
DOI 10.1111/ijac.13730
Issue 5
ISSN 1546-542X
Date Added 1/26/2024, 8:45:08 AM
Modified 1/26/2024, 8:45:08 AM

Survival and prosthetic complications of monolithic ceramic implant-supported single crowns and fixed partial dentures: A systematic review with meta-analysis

Type Journal Article

Author Cleidiel Aparecido Araujo Lemos
Author Fellippo Ramos Verri
Author Jéssica Marcela de Luna Gomes
Author Joel Ferreira Santiago Junior
Author Eduardo Miyashita
Author Gustavo Mendonça
Author Eduardo Piza Pellizzer
Date 2022-12
Publication The Journal of Prosthetic Dentistry
DOI 10.1016/j.prosdent.2022.11.013
ISSN 00223913
Date Added 1/26/2024, 8:45:06 AM
Modified 1/26/2024, 8:45:06 AM

Survival of zirconia- and metal-supported fixed dental prostheses: a systematic review.

Type Journal Article
Author Siegward D Heintze
Author Valentin Rousson
Abstract PURPOSE: The aim of this review was to systematically evaluate and compare the frequency of veneer chipping and core fracture of zirconia fixed dental prostheses (FDPs) and porcelain-fused-to-metal (PFM) FDPs and determine possible influencing factors. MATERIALS AND METHODS: The SCOPUS database and International Association of Dental Research abstracts were searched for clinical studies involving zirconia and PFM FDPs. Furthermore, studies that were integrated into systematic reviews on PFM FDPs were also evaluated. The principle investigators of any clinical studies on zirconia FDPs were contacted to provide additional information. Based on the available information for each FDP, a data file was constructed. Veneer chipping was divided into three grades (grade 1 = polishing, grade 2 = repair, grade 3 = replacement). To assess the frequency of veneer chipping and possible influencing factors, a piecewise exponential model was used to adjust for a study effect. RESULTS: None of the studies on PFM FDPs (reviews and additional searching) sufficiently satisfied the criteria of this review to be included. Thirteen clinical studies on zirconia FDPs and two studies that investigated both zirconia and PFM FDPs were identified. These studies involved 664 zirconia and 134 PFM FDPs at baseline. Follow-up data were available for 595 zirconia and 127 PFM FDPs. The mean observation period was approximately 3 years for both groups. The frequency of core fracture was less than 1% in the zirconia group and 0% in the PFM group. When all studies were included, 142 veneer chippings were recorded for zirconia FDPs (24%) and 43 for PFM FDPs (34%). However, the studies differed extensively with regard to veneer chipping of zirconia: 85% of all chippings occurred in 4 studies, and 43% of all chippings included zirconia FDPs. If only studies that evaluated both types of core materials were included, the frequency of chipping was 54% for the zirconia-supported FDPs and 34% for PFM FDPs. When adjusting the survival rate for the study effect, the difference between zirconia and PFM FDPs was statistically significant for all grades of chippings (P = .001), as well as for chipping grade 3 (P = .02). If all grades of veneer chippings were taken into account, the survival of PFM FDPs was 97%, while the survival rate of the zirconia FDPs was 90% after 3 years for a typical study. For both PFM and zirconia FDPs, the frequency of grades 1 and 2 veneer chippings was considerably higher than grade 3. Veneer chipping was significantly less frequent in pressed materials than in hand-layered materials, both for zirconia and PFM FDPs (P = .04). CONCLUSIONS: Since the frequency of veneer chipping was significantly higher in the zirconia FDPs than PFM FDPs, and as refined processing procedures have started to yield better results in the laboratory, new clinical studies with these new procedures must confirm whether the frequency of veneer chipping can be reduced to the level of PFM.
Date 2010
Volume 23
Pages 493–502
Publication The International journal of prosthodontics

**Issue**	6
**Date Added**	1/26/2024, 8:45:07 AM
**Modified**	1/26/2024, 8:45:07 AM

Tags:

chemistry, Dental Porcelain, Humans, Zirconium, Dental Polishing, Dental Restoration Failure, Dental Veneers, Denture, Denture Design, Denture Repair, Fixed, Metal Ceramic Alloys, Partial, Survival Analysis

---

Tensile bond strength of resin composite repair in vitro using different surface preparation conditionings to an aged CAD/CAM resin nanoceramic

**Type**	Journal Article
**Author**	Bogna Stawarczyk
**Author**	Andreas Krawczuk
**Author**	Nicoleta Ilie
**Date**	2015-03
**Volume**	19
**Pages**	299–308
**Publication**	Clinical Oral Investigations
**DOI**	10.1007/s00784-014-1269-3
**Issue**	2
**ISSN**	1432-6981
**Date Added**	1/26/2024, 8:45:03 AM
**Modified**	1/26/2024, 8:45:03 AM

The Consequences of Restorative Cycles

**Type**	Journal Article
**Author**	D. B. Henry
**Date**	2009-11
**Volume**	34
**Pages**	759–760
**Publication**	Operative Dentistry
**DOI**	10.2341/09-OP1
**Issue**	6
**ISSN**	1559-2863
**Date Added**	1/26/2024, 8:45:07 AM
**Modified**	1/26/2024, 8:45:07 AM

The Current State of Chairside Digital Dentistry and Materials

**Type**	Journal Article
**Author**	Markus B Blatz
**Author**	Julian Conejo
**Abstract**	This article describes and illustrates the current state of chairside computer-aided design computer-aided manufacturing technologies and materials. It provides a historical background and discusses the different components of the chairside digital workflow: intraoral scanners,

design software, milling machines, and sinter furnaces. The material range available for chairside digital dentistry is broad and includes polymethyl methacrylates, composite resins, and a large variety of ceramics. Clinical applications and success rates of the different material groups are summarized and discussed based on the current scientific evidence.

Date 2019
URL https://doi.org/10.1016/j.cden.2018.11.002
Extra Publisher: Elsevier Inc
Volume 63
Pages 175–197
Publication Dental Clinics of North America
DOI 10.1016/j.cden.2018.11.002
Issue 2
Date Added 1/26/2024, 8:45:08 AM
Modified 1/26/2024, 8:45:08 AM

Tags:

CAD/CAM, Ceramics, Chairside, Dental materials, Digital dentistry

---

The effect of different ceramic surface treatments on the repair bond strength of resin composite to lithium disilicate ceramic

Type Journal Article
Author Nanako Ueda
Author Tomohiro Takagaki
Author Toru Nikaido
Author Rena Takahashi
Author Masaomi Ikeda
Author Junji Tagami
Abstract This study aimed to evaluate the bonding abilities and long-term durability of different repair agents when applied to lithium disilicate ceramics (LDS). Blocks of IPS e.max CAD were prepared and divided into four groups according to the surface treatment: Monobond Etch & Prime (ME), K-etchant GEL+Clearfil Universal Bond (UB), Bondmer Lightless (BL), and K-etchant GEL+G-Multi Primer (GMP). All treated ceramic specimens were bonded to resin composite and light-cured. The micro-shear bond strength was measured after 24 h of water storage or 5,000 thermocycles. ME and BL showed significantly higher initial bond strengths than UB and GMP ($p<0.05$). After 5,000 thermocycles, there was no significant difference in ME ($p>0.05$), but BL and GMP showed considerable reduction in bond strength ($p<0.05$); moreover, the samples of UB were all de-bonded before testing. ME and BL proved to be effective surface treatment materials for LDS.
Date 2021
Extra Publisher: Japanese Society for Dental Materials and Devices PMID: 33883329
Volume 40
Pages 1073–1079
Publication Dental Materials Journal
DOI 10.4012/dmj.2020-362
Issue 5
ISSN 18811361
Date Added 1/26/2024, 8:45:04 AM
Modified 1/26/2024, 8:45:04 AM

Tags:

Lithium disilicate ceramic, Micro-shear bond strength, Repair, Silane, Thermocycling

---

The Effect of Multiple Applications of Phosphate-Containing Primer on Shear Bond Strength between Zirconia and Resin Composite

Type Journal Article
Author Awiruth Klaisiri
Author Apichai Maneenacarith
Author Nicha Jirathawornkul
Author Panattha Suthamprajak
Author Tool Sriamporn
Author Niyom Thamrongananskul
Abstract <p>Occasional chipping can still occur with zirconia material despite its high strength. Emergency repairs can be accomplished using zirconia primer, adhesive agent, and resin composite when the fracture of zirconia exposes the zirconia framework. Phosphate-containing primers play an important role in zirconia surface treatment. The objective of this investigation was to evaluate the effect of multiple applications of phosphate-containing primer on shear bond strength between zirconia and resin composite. In this case, 78 zirconia discs were sandblasted by alumina particles; the zirconia was then randomized into six groups for single application and multiple applications of phosphate-containing primer according follows; group 1: no application, group 2: one application, group 3: two applications, group 4: three applications, group 5: four applications, and group 6: five applications. Adhesive was applied on the zirconia surface and the resin composite was bonded. Shear bond strength was assessed using a universal testing machine. The de-bonded surface was examined using a stereomicroscope. The shear bond strengths were statistically analyzed with one-way ANOVA and Bonferroni. Group 1 had the lowest shear bond strength with a significant difference compared to groups 2–6, whereas group 4 had the highest shear bond strength, with no significant difference compared to groups 5–6. The failure mode revealed 100% adhesive failure in all groups. In conclusion, to maximize shear bond strength at zirconia and resin composite interfaces, sandblasted zirconia surfaces should be treated with three applications of phosphate-containing primer prior to the adhesive agent.</p>
Date 2022-10
Volume 14
Pages 4174
Publication Polymers
DOI 10.3390/polym14194174
Issue 19
ISSN 2073-4360
Date Added 1/26/2024, 8:45:07 AM
Modified 1/26/2024, 8:45:07 AM

The effect of repeated firings on the color change and surface roughness of dental ceramics

Type Journal Article
Author Fehmi Gonuldas
Author Kerem Yılmaz
Author Caner Ozturk
Date 2014
Volume 6
Pages 309

Publication The Journal of Advanced Prosthodontics
DOI 10.4047/jap.2014.6.4.309
Issue 4
ISSN 2005-7806
Date Added 1/26/2024, 8:45:08 AM
Modified 1/26/2024, 8:45:08 AM

The Effect of Resin Bonding on Long-Term Success of High-Strength Ceramics

Type Journal Article
Author M B B Blatz
Author M Vonderheide
Author J Conejo
Abstract Digital manufacturing, all-ceramics, and adhesive dentistry are currently the trendiest topics in clinical restorative dentistry. Tooth- and implant-supported fixed restorations from computer-aided design (CAD)/computer-aided manufacturing (CAM)–fabricated high-strength ceramics—namely, alumina and zirconia—are widely accepted as reliable alternatives to traditional metal-ceramic restorations. Most recent developments have focused on high-translucent monolithic full-contour zirconia restorations, which have become extremely popular in a short period of time, due to physical strength, CAD/CAM fabrication, and low cost. However, questions about proper resin bonding protocols have emerged, as they are critical for clinical success of brittle ceramics and treatment options that rely on adhesive bonds, specifically resin-bonded fixed dental prostheses or partial-coverage restorations such as inlays/onlays and veneers. Resin bonding has long been the gold standard for retention and reinforcement of low- to medium-strength silica-based ceramics but requires multiple pretreatment steps of the bonding surfaces, increasing complexity, and technique sensitivity compared to conventional cementation. Here, we critically review and discuss the evidence on resin bonding related to long-term clinical outcomes of tooth- and implant-supported high-strength ceramic restorations. Based on a targeted literature search, clinical long-term studies indicate that porcelain-veneered alumina or zirconia full-coverage crowns and fixed dental prostheses have high long-term survival rates when inserted with conventional cements. However, most of the selected studies recommend resin bonding and suggest even greater success with composite resins or self-adhesive resin cements, especially for implant-supported restorations. High-strength ceramic resin-bonded fixed dental prostheses have high long-term clinical success rates, especially when designed as a cantilever with only 1 retainer. Proper pretreatment of the bonding surfaces and application of primers or composite resins that contain special adhesive monomers are necessary. To date, there are no clinical long-term data on resin bonding of partial-coverage high-strength ceramic or monolithic zirconia restorations.
Date 2018
URL https://pubmed.ncbi.nlm.nih.gov/28876966/
http://journals.sagepub.com/doi/10.1177/0022034517729134
Extra Publisher: SAGE Publications Inc.
Volume 97
Pages 132–139
Publication Journal of Dental Research
DOI 10.1177/0022034517729134
Issue 2
ISSN 0022034517729
Date Added 1/26/2024, 8:45:08 AM
Modified 1/26/2024, 8:45:08 AM

Tags:

adhesives, CAD, cement, clinical outcomes, esthetic dentistry, prosthetic dentistry/prosthodontics

The Effect of Surface Treatments on the Bond Strength Between CAD/CAM Blocks and Composite Resin

Type Journal Article
Author Mustafa Duzyol
Author Omer Sagsoz
Author Nurdan Polat Sagsoz
Author Nilgun Akgul
Author Mehmet Yildiz
Abstract Purpose: The aim of this study was to evaluate the microtensile bond strength of three computer-aided design/computer-aided manufacturing (CAD/CAM) blocks repaired with composite resin using three surface treatment techniques. Methods and Materials: Three different CAD/CAM blocks were used in this study: (1) Lithium disilicate, (2) feldspar ceramic, and (3) resin nano ceramic. All groups were further divided into four subgroups according to surface treatment: control, roughened with bur (B); roughened with bur and 5% Hydrofluoric acid (HF); roughened with bur and sandblasting (HF); and roughened with bur and CoJet (C). After surface treatments on each group, a silane and bonding agent were applied, and ceramics were repaired with a nano-hybrid composite. Then, the repaired ceramics were cut with a low-speed diamond saw for microtensile bond testing. Microtensile bond tests for 40 specimens per subgroup were carried out with a universal testing machine. The data were analyzed with ANOVA, Tukey's, and LSD at the 95% significance level. Results: Mean bond strengths (MPa) of subgroups B, HF, S, and C were: 0, 29.8, 0, 23.3 for lithium disilicate ceramic; 26.4, 22.3, 22.4, 22 for feldspar ceramic; 54.8, 25.3, 42.1, 25.7 for resin nano ceramic. For subgroups B and S of lithium disilicate ceramics, bonding failed during specimen preparation. No significant differences were observed among all CoJet groups. In subgroups B and S, resin nano ceramics showed the highest bond strength. In feldspar groups, subgroup B showed higher bond strength than the other subgroups. Conclusion: This study demonstrates that lithium disilicate porcelain blocks required etching for repairing with composite material. Surface treatments did not increase the bond strength in feldspar ceramic groups and reduced the bond strength in resin nano ceramic groups.
Date 2016
Extra Publisher: Blackwell Publishing Inc. PMID: 26216441
Volume 25
Pages 466–471
Publication Journal of Prosthodontics
DOI 10.1111/jopr.12322
Issue 6
ISSN 1532849X
Date Added 1/26/2024, 8:45:08 AM
Modified 1/26/2024, 8:45:08 AM

Tags:

acid etching, CAD/CAM restorations, intraoral porcelain repair, microtensile bond strength, resin composite, sandblasting, surface treatment

Three generations of zirconia: From veneered to monolithic. Part I.

Type Journal Article
Author Bogna Stawarczyk
Author Christin Keul
Author Marlis Eichberger
Author David Figge
Author Daniel Edelhoff

Author Nina Lümkemann

Abstract This article presents the historical development of the different generations of zirconia and their range of indications, from veneered to monolithic zirconia restorations. Because of the large extent of this topic, it is divided into two parts. In Part I, the mechanical and optical properties of the three generations of zirconia materials are discussed critically and theoretically. A short summary is given of the current scientific literature, investigating the third generation of zirconia comparatively regarding the properties discussed.

Date 2017

Extra PMID: 28396886

Volume 48

Pages 369–380

Publication Quintessence international (Berlin, Germany : 1985)

DOI 10.3290/j.qi.a38057

Issue 5

ISSN 1936-7163

Date Added 1/26/2024, 8:45:05 AM

Modified 1/26/2024, 8:45:05 AM

Unstable cracking (chipping) of veneering porcelain on all-ceramic dental crowns and fixed partial dentures

Type Journal Article

Author M.V. Swain

Date 2009-06

Volume 5

Pages 1668–1677

Publication Acta Biomaterialia

DOI 10.1016/j.actbio.2008.12.016

Issue 5

ISSN 17427061

Date Added 1/26/2024, 8:45:05 AM

Modified 1/26/2024, 8:45:05 AM

Which surface treatment promotes higher bond strength for the repair of resin nanoceramics and polymer-infiltrated ceramics? A systematic review and meta-analysis

Type Journal Article

Author Dayanne Monielle D. Moura

Author Aretha Heitor Veríssimo

Author Taciana Emília Leite Vila-Nova

Author Patrícia Santos Calderon

Author Mutlu Özcan

Author Rodrigo Othávio Assunção Souza

Date 2022-08

Volume 128

Pages 139–149

Publication The Journal of Prosthetic Dentistry

DOI 10.1016/j.prosdent.2020.06.009

Issue 2

ISSN 00223913

Date Added 1/26/2024, 8:45:06 AM
Modified 1/26/2024, 8:45:06 AM
